# Tissue Distribution of Total Flavonoids Extracts of *Drynariae Rhizoma* in Young and Old Rats by UPLC–MS/MS Determination

**DOI:** 10.1155/2022/2447945

**Published:** 2022-02-04

**Authors:** Yue Zhang, Xia Lei, Hongdan Xu, Guoliang Liu, Yeqiu Wang, Huifeng Sun, Fang Geng, Ning Zhang

**Affiliations:** ^1^College of Pharmacy, Heilongjiang University of Chinese Medicine, Harbin, Heilongjiang 150040, China; ^2^College of Jiamusi, Heilongjiang University of Chinese Medicine, Jiamusi, Heilongjiang 154007, China; ^3^College of Chemistry & Chemical Engineering, Harbin Normal University, Harbin, Heilongjiang 150025, China; ^4^Key Laboratory of Chinese Materia Medica (Heilongjiang University of Chinese Medicine), Ministry of Education, Harbin, Heilongjiang 150025, China

## Abstract

*Drynariae Rhizoma* (Kunze ex Mett.) J. Sm. has been extensively used in China, Japan, and Korea to treat osteoporosis and tonify kidneys. A rapid and validated UPLC-MS/MS method for simultaneous determination of the seven analytes including neoeriocitrin, luteolin-7-*O-β*-D-glucoside, astragalin, naringin, eriodictyol, naringenin, and kaempferol in rats' various tissues (heart, liver, spleen, lung, kidney, stomach, brain, uterus, ovary, and small intestine) using quercetin as the internal standard (IS) was developed after oral administration of TFDR to rats. Tissues samples were retreated by protein precipitation with methanol. The chromatographic separation was performed using an ACQUITY UPLC™ BEH C_18_ column (2.1 × 50 mm; 1.7 *μ*m) at 35°C. The mobile phase consisted of 1% acetic acid in water as the aqueous phase (A) and 100% acetonitrile as the organic phase (B). All analytes and IS were quantified through electrospray ionization in positive ion multiple reaction monitoring (MRM) mode. MS transitions were m/z 597.5 ⟶ 289.2 for neoeriocitrin, m/z 449.1 ⟶ 287.1 for luteolin-7-*O-β*-D-glucoside, m/z 449.1 ⟶ 287.1 for astragalin, m/z 581.5 ⟶ 273.2 for naringin, m/z 289.2 ⟶ 153.1 for eriodictyol, m/z 273.2 ⟶ 153.1 for naringenin, m/z 287.1 ⟶ 153.1 for kaempferol, and m/z 303.2 ⟶ 153.1 for quercetin (IS). The mean extraction recovery of the seven analytes and IS in tissue samples at three levels of quality control (QC) samples ranged from 82.72% to 118.57%, and the RSD was ≤14.98%. The intraday and interday precision (RSD) were all less than 14.98%, and the accuracy (RE) ranged from −13.96% to 14.96%, which indicated that the present method was not an issue. Tissues distribution showed neoeriocitrin, luteolin-7-*O-β*-D-glucoside, astragalin, naringin, and naringenin could transfer across the blood-brain barrier, which may form the basis of TFDR entering the brain to play an anti-AD role. Compared with the 8-month-old rats, a higher concentration of naringin was found in the ovaries of the 18-month-old rats; this result indicated that it may regulate the autonomic nervous dysfunction of the cerebrospinal system through the hypothalamus-pituitary-ovary axis, thus playing an anti-AD role, but further research is needed. Naringenin, eriodictyol, and kaempferol have a higher concentration in the liver and kidney; this phenomenon may be related to the traditional Chinese medicine theory that there is a definite relationship between the liver and kidney meridian. These results provide reliable data support for further study of the pharmacological mechanism of TFDR, formulation of drug delivery schemes, and development of new Chinese medicines in the treatment of AD.

## 1. Introduction

**Figure 1:**
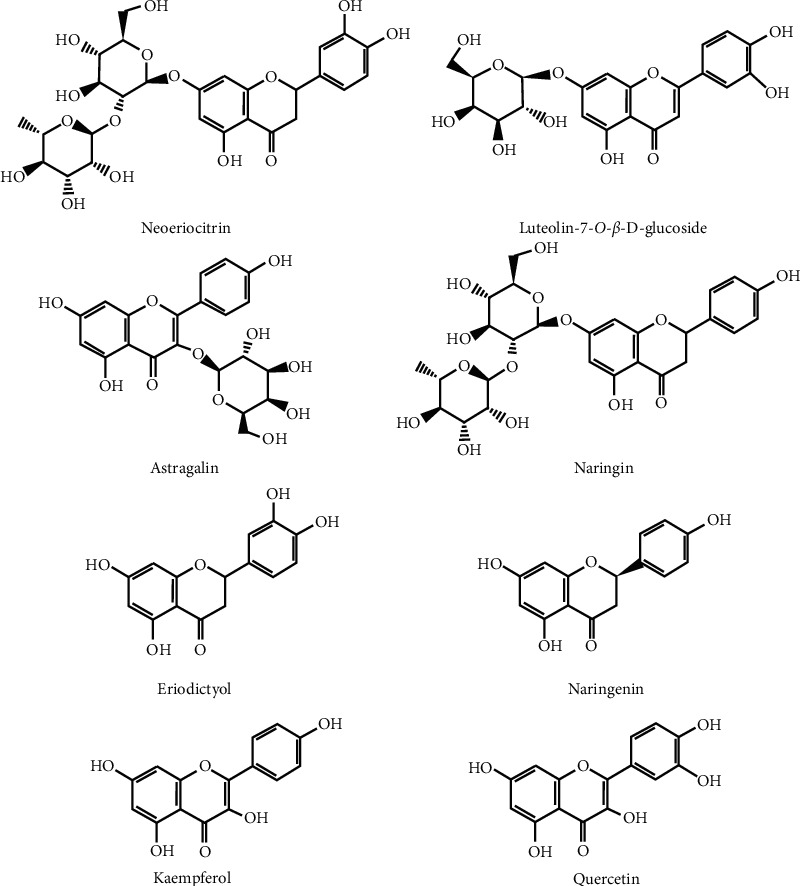
Chemical structures of seven analytes and IS.

Alzheimer's disease (AD) is a kind of disease that leads to the decline of human memory and affects the cognitive function of the brain, which seriously threatens human life and health [1, 2]. According to traditional medicine, the main system of Alzheimer's disease is the deficiency of kidney qi, leading to insufficient marrow sea; thus, the brain tissue atrophy and functional decline. Therefore, herbal medicine possessing the efficacy of nourishing kidney essence or replenishing brain marrow are commonly served as effective strategies for AD treatment [3–7]. *Drynariae Rhizoma* is a common plant widely distributed in southern China [8]. Total *Drynariae Rhizoma* flavonoids is a classic Chinese herbal medicine that contains mainly flavonoids and is prepared based on the China Pharmacopoeia standard of quality control [9]. Total *Drynariae Rhizoma* flavonoids have been used in the clinic to nourish the kidneys, strengthen the bones, cure injuries, and relieve pains [10]. Studies have revealed that total flavonoids from *Drynariae Rhizoma* (TFDR) could promote fracture healing, and the mechanism is mainly delineated by improving blood circulation, relieving blood flow abnormalities, and preventing blood clots [11]. *Drynariae Rhizoma* is a classic Chinese medicine for invigorating the kidney and strengthening the bones, which is consistent with the treatment of Alzheimer's disease from the perspective of the basic theory of traditional Chinese medicine, and modern pharmacology shows that *Drynaria fortunei* has the function of improving learning and memory, and its main component is flavonoids [12, 13]. TFDR can activate estrogen receptors and has the tendency to replace estrogens in clinical practice [14]. In our previous study, using 55% ethanol reflux extraction and AB-8 macroporous resin as carrier, the total flavonoids content of *Rhizoma Drynariae* was 76.11%, in which naringin content was 20.41%. In the study of the protective effect of TFDR on PC12 cells induced by A*β*25-35, TFDR can effectively inhibit the death of PC12 cells induced by A*β*25-35 [15]. In the analysis of blood components and pharmacokinetics of SD rats fed with Gufangbu flavonoids by gavage, neoeriocitrin, luteolin-7-*O-β*-D-glucoside, astragalin, naringin, eriodictyol, luteolin, naringenin, and kaempferol (Figure 1) are thought as the active components of TFDR [16]. As we all know, pharmacokinetics studies could help greatly understand and confirm the efficacy and action mechanism of drugs as well as optimize benefit and reduce harm. There are many ingredients in TCM; not just a single component plays a role in TCM completely, but the outcome of many components combing action does. Until now, few studies have concentrated on tissues distribution study of these bioactive compounds in *Drynariae Rhizoma*.

In this study, a rapid and validated UPLC-MS/MS method for simultaneous determination of neoeriocitrin, luteolin-7-*O-β*-D-glucoside, astragalin, naringin, eriodictyol, naringenin, and kaempferol in rat tissue was successfully applied after oral administration of TFDR to rats. The study would also promote to safely and reasonably use *Drynariae Rhizoma* in the clinic.

## 2. Experimental Section

### 2.1. Chemicals, Reagents, and Analytical Conditions

The research content of this experiment is an in-depth study of the article “Pharmacokinetics of Eight Flavonoids in Rats Assayed by UPLC-MS/MS after Oral Administration of *Drynariae rhizoma* Extract” published in 2018. The preparation, quality evaluation, and analysis conditions of chemicals, reagents, and extracts are based on the experimental methods established in the early stage of the laboratory. See reference [16] for details.

### 2.2. Preparation of Calibration Standards and Quality Control Samples

Each reference compound was accurately weighed and dissolved in methanol/water (50/50, v/v) as a primary stock solution at the concentration of 1 mg/mL and was prepared as a series of standard or QC solutions at the desired concentrations by serial dilution. The IS working solution of 200 ng/mL was prepared by dilution of the stock standard solution with methanol/water (50/50, v/v). Different tissues calibration standard solutions were prepared at different concentrations for eight different flavonoids. Three concentrations (low, middle, and high concentrations) of each analyte solution in drug-free tissue were used for quality control (QC) evaluation in UPLC-MS/MS analysis. All the solutions were stored at 4°C and brought to room temperature before use.

### 2.3. Pretreatment of Tissue Samples

For tissue homogenate sample preparation, each weighed 0.2 g tissue sample was thawed, cut up into a 2 ml tube, and then homogenized in ice-cold physiological saline (5 ml/g tissue). Then 500 *μ*l of tissue homogenate sample was mixed with 2 ml methanol containing 50 *μ*l of internal standard solution (200 ng/ml) and 50 *μ*l acetic acid in a 10 ml centrifuge tube. The mixture was then vortex-mixed for 2 min for the purpose of protein precipitation. After centrifuging at 14,000 ×g for 10 min, the supernatant was transferred into another centrifuge tube. The vortexing and centrifugation process was then repeated, and the two supernatants were combined and evaporated to dryness under a flow of nitrogen gas at 40°C. The residue was then dissolved in 100 *μ*L methanol and vortexed for 1 min. Finally, the vortexing and centrifugation process was again repeated, and 5 *μ*L of the supernatant was injected into the UPLC-MS/MS system for analysis.

### 2.4. Assay Validation

#### 2.4.1. Selectivity

The selectivity of the method was determined by analyzing the MRM chromatograms of six different blank tissues homogenate samples, blank tissue homogenate spiked with seven analytes, and IS and tissue homogenate after administration of TFDR.

#### 2.4.2. Calibration Curves and Lower Limit of Quantitation (LOQ)

Calibration curves of analytes were prepared by adding a series of different concentration working solutions and IS working solutions to tissues homogenate to determine the linearity and LOQ and assessed by weighted least-squares linear regression using 1/*x*^2^ as a weighting factor.

#### 2.4.3. Precision and Accuracy

Intraday and interday precision and accuracy were determined by analyzing six replicates at three different QC samples (low, middle, and high concentrations).

#### 2.4.4. Extraction Recovery and Matrix Effect

The extraction recovery was determined by comparing the peak areas between tissues homogenate samples spiked with analytes before and after extraction for low, middle, and high concentration QC samples with six replicates. The matrix effect was evaluated by comparing the peak areas between the low, middle, and high concentration QC samples spiked in blank tissue homogenate and pure standard solutions at the same concentrations with six replicates.

#### 2.4.5. Stability

The stability of analytes was tested under different storage and process conditions for low, middle, and high concentrations QC samples with six replicates: store auto-sampler (4°C) for 36 h, keep in −20°C for 30 days, and perform three complete freeze/thaw cycles from −20°C to room temperature.

### 2.5. Tissue Distribution Study

#### 2.5.1. Animals

Fifty-four 8-month-old and 18-month-old female Sprague-Dawley rats (280–350 g) were supplied by the Animal Safety Evaluation Center of Heilongjiang University of Chinese Medicine (Harbin, China). The rats were housed in an air-conditioned room at a temperature of 22 ± 2°C and relative humidity of 50 ± 10% with 12 h dark-light cycles and allowed food and water spontaneously. All protocols of animal experiments were approved in accordance with the regulations of Experimental Animal Administration issued by the Animal Ethics Committee of the institution.

#### 2.5.2. Drug Administration and Sampling

Fifty-five 8-month-old and 18-month-old rats were randomly divided into 6 groups (9 rats per group), respectively. Before oral administration, rats were fasted overnight but allowed access to water freely. After oral administration of TFDR at a dose of 4 g/kg to each rat, tissues (heart, liver, spleen, lung, kidney, stomach, brain, uterus, ovary, and small intestine) were collected at 0, 0.25, 0.5, 1, 2, 4, 8, 12, and 24 h. Tissue samples were rinsed with physiological saline to remove the blood, then blotted on filter paper, and stored at −80°C until analysis.

## 3. Results and Discussion

### 3.1. LC-MS Conditions

The molecular weight, parent ion, daughter ion, cone voltage, collision energy, and retention time of the seven flavonoids and IS are shown in Table 1.

### 3.2. Assay Validation

#### 3.2.1. Selectivity

**Figure 2:**
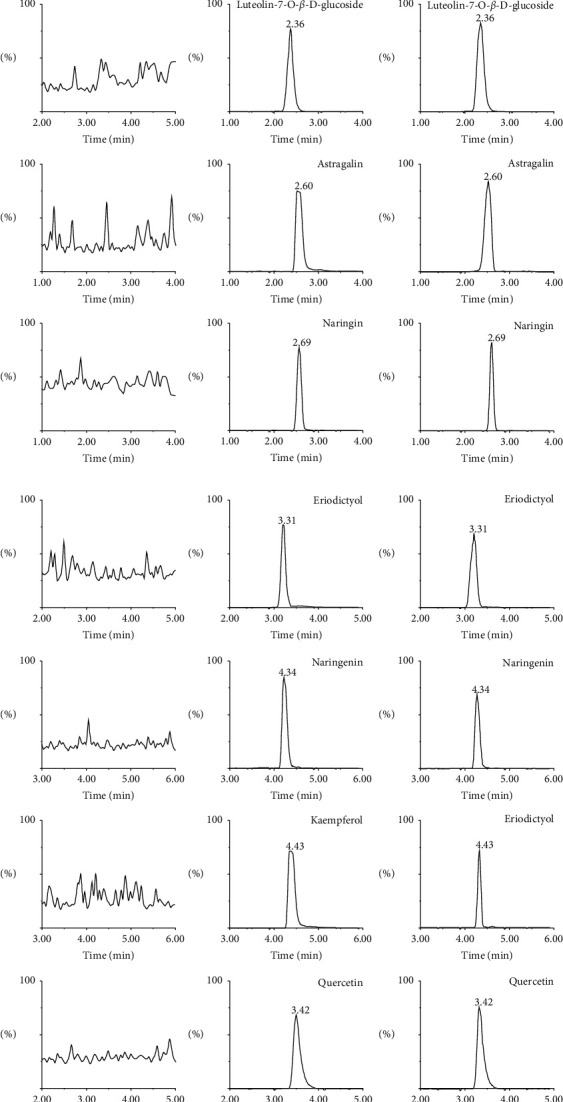
Representative MRM chromatograms of seven analytes and quercetin (IS): (a) blank liver homogenates; (b) blank liver homogenates spiked with the seven analytes and IS; and (c) liver homogenates 1 h after oral administration of TFDR (4 g/kg; mean ± SD; *n* = 6).

The chromatograms of blank tissues homogenate sample, blank tissues homogenate sample spiked with seven analytes and IS, and tissue homogenate after administration of TFDR are shown in Figure 2. No interfering from endogenous components was observed in the retention time of the seven analytes and IS, which demonstrated an acceptable selectivity in the conditions.

#### 3.2.2. Calibration Curves and Lower Limit of Quantitation (LOQ)

The regression equation, correlation coefficient, and linear ranges of the seven analytes in various tissues are listed in Table 2. In the detection of tissue homogenate samples, correlation coefficients (*R*^2^) of calibration curves were greater than 0.999, showed good linearity over the concentration ranges for the seven analytes. The LOQ in various tissues is presented in Table 2. The present LOQ with a signal-to-noise ratio >10 was sensitive enough to investigate seven analytes after oral administration of TFDR.

#### 3.2.3. Precision and Accuracy

The intraday and interday precision and accuracy of the seven analytes in the various tissues at three different QC levels (low, middle, and high) were estimated and are shown in Tables 3–9. The intraday and interday precision (RSD) were all less than 14.98%, and the accuracy (RE) ranged from −13.96% to 14.96%, which indicated that the present method was not a problem.

#### 3.2.4. Extraction Recovery and Matrix Effect

The mean extraction recovery of the seven analytes and IS in tissue samples at three levels of QC samples ranged from 82.72% to 118.57%, and the RSD was ≤14.98%. The matrix effect value was obtained for the seven analytes and IS ranged from 84.95% to 117.51% within 14.98% RSD. The results in Tables 10–16 suggested that the extraction recovery and matrix effect for the seven analytes and IS were in the acceptable range, and there is no interference from endogenous components.

#### 3.2.5. Stability

The stability of the seven analytes in tissue samples at three different levels of QC samples was performed according to the procedure described above. Results of stability studies shown in Tables 17–23 indicate that the seven analytes in tissue samples were stable when stored in auto-sampler (4°C) for 36 h, kept in −20°C for 30 days, and performed three complete freeze/thaw cycles from −20°C at room temperature. No significant degradation occurred during any of the experiment.

### 3.3. Tissue Distribution Study

**Figure 3:**
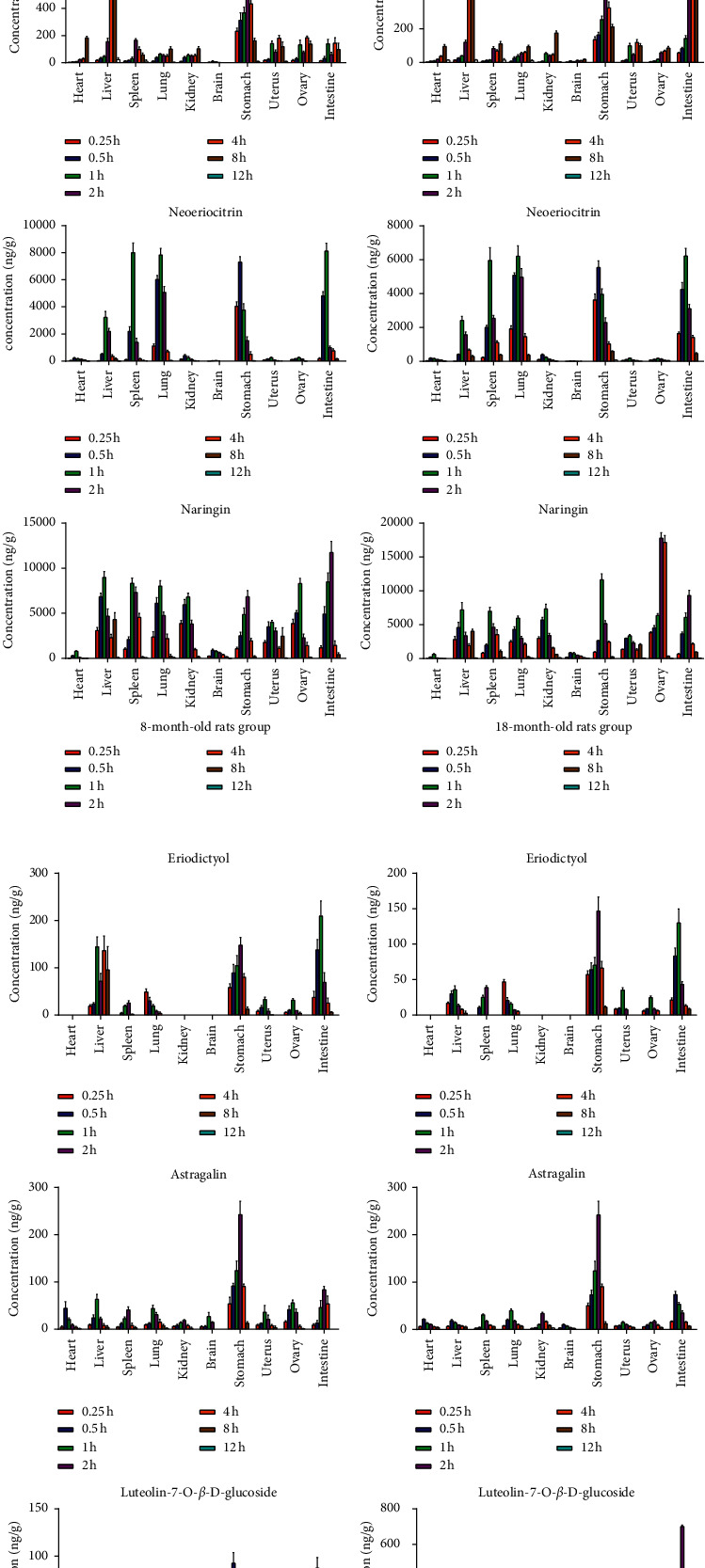
Concentrations of seven analytes in rat tissues at 0.25, 0.5, 1, 2, 4, 8, and 12 h after oral administration of TFDR in 8-month-old rats (left) and 18-month-old rats (right).

The tissue distribution of the seven analytes was investigated in 8-month-old and 18-month-old SD rats at 0, 0.25, 0.5, 1, 2, 4, 8, 12, and 24 h after oral administration of TFDR by collecting tissues including the heart, liver, spleen, lung, kidney, stomach, brain, uterus, ovary, and small intestine. The tendency graphs of the seven analytes in various SD rat tissues are shown in Figure 3. The concentrations of seven analytes in 8-month-old and 18-month-old rats' various tissues after oral administration of TFDR are shown in Tables 24 and 25, respectively. Luteolin was not detected in tissues. It may be that the concentration of luteolin in tissues was too low to reach the limit of detection. The results of the present study indicated that seven analytes underwent a rapid and wide distribution into tissues.

Neoeriocitrin, luteolin-7-*O-β*-D-glucoside, astragalin, naringin, and naringenin are distributed in the heart, liver, spleen, lung, kidney, brain, stomach, uterus, ovary, and small intestine. Eriodictyol was not detected in the heart, kidney, and brain but was distributed in other tissues; kaempferol was not detected in the heart, brain, and ovary and was distributed to other tissues.

After oral administration of TFDR in rats, the highest tissue concentration of neoeriocitrin was detected in the intestine (8,133.84 ng/g), followed by the spleen (8,008.91 ng/g), the lung (7,837.09 ng/g), the stomach (7,324.59 ng/g), and the liver (3,247.80 ng/g) in 8-month-old rats. However, the highest tissue concentration of neoeriocitrin was detected in the intestine (6,236.26 ng/g), followed by the lung (6,223.83 ng/g), the spleen (5,970.17 ng/g), the stomach (5,552.27 ng/g), and the liver (2,431.18 ng/g) in 18-month-old rats. From the results, we found that the maximum concentration of neoeriocitrin was observed in the intestine, which may be mainly attributed to the oral mode of administration. The concentration of total neoeriocitrin in the lung was significantly higher than that in other tissues in rats, demonstrating that neoeriocitrin was mainly accumulated in the lung suggesting a potential role of this organ in the metabolism of neoeriocitrin. The total neoeriocitrin exhibited a litter lower concentration in the heart, liver, kidney, brain, uterus, and ovary, which indicate that blood flow and the organ did not play a key role in the distribution of neoeriocitrin. In addition, neoeriocitrin could be detected in the brain, which illustrated that could transfer across the blood-brain barrier, which may be related to the treatment of brain-related diseases, and further study is necessary.

Luteolin-7-*O-β*-D-glucoside and astragalin are mainly distributed into the digestive system of the rat's body after oral administration of TFDR. In the timescale, the peak concentration of luteolin-7-*O-β*-D-glucoside and astragalin appeared at different time points for the different tissues in 8-month-old and 18-month-old rats. The highest concentration was found in the stomach at 0.5 h in 8-month-old rats, while for 18-month-old rats, it was detected in the intestine at 2 h. For astragalin, the highest concentration was found in the stomach at 2 h in 8-month-old and 18-month-old rats. After 2 h, levels of luteolin-7-*O-β*-D-glucoside and astragalin in all tissues went down with the time. The distribution of luteolin-7-*O-β*-D-glucoside and astragalin in the brain showed that it had the ability to cross the blood-brain barrier after oral administration, which may be related to the treatment of brain-related diseases, and further research is still needed. It was showed that luteolin-7-*O-β*-D-glucoside and astragalin were mainly distributed in the stomach and intestine, which implied the stomach may be the major organ to absorb them.

Naringin, as the highest abundant flavonoids in TFDR, shows a wide distribution in all the tissues, and a small amount also crosses the blood-brain barrier. Compared with other tissues, it was less distributed in the heart and brain of rats. Tissue-to-plasma AUC ratio (T/P ratio) suggested that the maximum amount of drug was found in the intestine and liver; this finding was expected when the drug has given by oral administration and when it has major first-pass metabolism in the liver and intestine [17, 18]. However, it was more distributed in stomach, intestine, and ovary in old rats; this result indicated that it may regulate the autonomic nervous dysfunction of the cerebrospinal system through the hypothalamus-pituitary-ovary axis, thus playing an anti-AD role, but further research is needed [19].

The peak concentration of naringenin, the aglycone of naringin, was observed in the stomach at 2 h in 8-month-old and 18-month-old rats, followed by intestine at 4 h, and decreased gradually with time in tissues; this indicated that naringenin had strong selectivity to liver and kidney. Naringenin is slowly absorbed in liver and kidney tissues and has a long action time, which may be due to the metabolism of naringin into naringenin in vivo [20, 21]. It is speculated that this phenomenon may be related to the traditional Chinese medicine theory that there is a certain relationship between the liver and kidney meridian.

The peak concentration of eriodictyol was observed in the liver tissue; this phenomenon may be related to the traditional Chinese medicine theory that there is a certain relationship between the liver and kidney meridian. It could hardly be detected in the heart and brain.

Kaempferol is also widely and rapidly distributed into various tissues. There was more distribution in the spleen of rats at 1 h, indicating that kaempferol has a strong affinity with the spleen. There was more distribution in the kidney of rats at 2 h. The reason for the higher content of kidney tissue may be that the kidney is the main metabolic organ of the drug, and most of the drugs are excreted through the kidney. It could hardly be detected in the heart and brain [22].

According to the tissue distribution of seven compounds in the total *Drynariae Rhizoma* flavonoids in the young group and the old group, it was found that naringenin was rapidly absorbed in the stomach. However, naringin and neoeriocitrin were hydrolyzed into aglycons or absorbed in the belly of rats. The glycosides connected with one sugar at *R*_3_ (neoeriocitrin, eriodictyol, and luteolin-7-*O-β-D*-glucoside) are mainly distributed in the small intestine. Few tests in the kidney indicate that they may be transformed through the liver and discharged into the small intestine from the bile, converted into prototype drugs by corresponding hydrolases, and reabsorbed. It can be seen that the structure of the mother nucleus of flavonoids and the number and location of linked glycosyls will affect the tissue distribution. Clarifying the mechanism of these interactions is of great significance to increase the safety of the clinical medication and promote the development and clinical application of flavonoids.

This is the first study to examine the tissue distribution profile of multiple bioactive components after oral administration of TFDR. The results of the tissue distribution study showed that the seven analytes were of TFDR distributed mainly into the gastrointestinal tract, which provided the material basis for its pharmacological actions in clinical application.

## 4. Conclusions

In this study, a rapid and validated UPLC-MS/MS method for simultaneous determination of the seven analytes in rat tissue was successfully applied after oral administration of TFDR to rats. To the best of our knowledge, this is the first report of the UPLC-MS/MS method on the tissue distribution of the seven analytes in rats. The tissue distribution results achieved might be useful for further study of the bioactive mechanism of *Drynariae Rhizoma*. The validation procedure confirmed that the method was sensitive and selective and was suitable for the analysis of the seven analytes in biological samples. The present study could promote understanding the distribution of TFDR in vivo and provide the material basis for the dose regimen in the clinic.

## Figures and Tables

**Table 1:** Precursor ion and product ion transition and parameters of the analytes used in this study.

Analytes	Retention time (min)	Precursor ion species	MRM transition		Cone (V)	Collision (V)	Dwell time (ms)
			Precursor ion	Product ion			
Neoeriocitrin	2.20	[M+H]+	597.5	289.2	25	30	0.2
Luteolin-7-*O-β*-D-glucoside	2.36	[M+H]+	449.1	287.1	20	20	0.2
Astragalin	2.60	[M+H]+	449.1	287.1	30	30	0.2
Naringin	2.69	[M+H]+	581.5	273.2	20	25	0.2
Eriodictyol	3.31	[M+H]+	289.2	153.1	30	25	0.2
Naringenin	4.34	[M+H]+	273.2	153.1	20	30	0.2
Kaempferol	4.43	[M+H]+	287.1	153.1	30	30	0.2
Quercetin (IS)	3.42	[M+H]+	303.2	153.1	20	25	0.2

**Table 2:** The linear equations and range of seven analytes in different rats' tissues.

Analytes	Tissue	Regression equation	Correlation coefficient	Concentration range (ng/mL)	Standard error (slope)	Standard error (intercept)
Neoeriocitrin	Heart	*y* = 0.9013*x* + 5.1928	*R* ^2^ = 0.9991	5–500	4.0987	0.0146
	Liver	*y* = 0.0972*x* + 36.321	*R* ^2^ = 0.9991	10–5,000	3.8552	0.0014
	Spleen	*y* = 0.0931*x* + 11.703	*R* ^2^ = 0.9997	5–10,000	4.6785	0.0008
	Lung	*y* = 0.1685*x* + 12.194	*R* ^2^ = 0.9990	10–10,000	5.9655	0.4775
	Kidney	*y* = 1.195*x* + 5.4235	*R* ^2^ = 0.9997	5–500	4.0379	0.0144
	Brain	*y* = 2.7542*x* − 1.5057	*R* ^2^ = 0.9991	5–100	2.5959	0.0464
	Stomach	*y* = 0.1753*x* + 20.86	*R* ^2^ = 0.9992	5–10,000	10.5855	0.0023
	Uterus	*y* = 1.5008*x* − 4.2356	*R* ^2^ = 0.9991	5–500	7.4003	0.0265
	Ovary	*y* = 1.8168*x* − 7.1718	*R* ^2^ = 0.9994	5–500	7.3924	0.0264
	Intestine	*y* = 0.0948*x* + 22.078	*R* ^2^ = 0.9992	5–10,000	5.9586	0.0013
Luteolin-7-*O-β*-D-glucoside	Heart	*y* = 17.011*x* − 11.593	*R* ^2^ = 0.9996	2–50	5.3807	0.1925
	Liver	*y* = 14.803*x* − 5.3733	*R* ^2^ = 0.9996	2–50	4.6633	0.1668
	Spleen	*y* = 19.649*x* − 27.686	*R* ^2^ = 0.9992	2–50	8.9679	0.3209
	Lung	*y* = 27.742*x* + 144.06	*R* ^2^ = 0.9990	5–100	18.3343	0.5063
	Kidney	*y* = 22.7*x* − 30.98	*R* ^2^ = 0.9992	2–50	10.4027	0.3722
	Brain	*y* = 17.311*x* − 0.2042	*R* ^2^ = 0.9995	2–50	6.0981	0.2182
	Stomach	*y* = 25.13*x* − 45.82	*R* ^2^ = 0.9997	5–100	14.4218	0.2579
	Uterus	*y* = 22.459*x* − 27.379	*R* ^2^ = 0.9993	2–50	9.7807	0.3500
	Ovary	*y* = 15.526*x* − 28.948	*R* ^2^ = 0.9995	2–50	5.2243	0.1869
	Intestine	*y* = 23.444*x* − 62.646	*R* ^2^ = 0.9990	5–100	3.2885	0.4166

Astragalin	Heart	*y* = 33.567*x* − 145.2	*R* ^2^ = 0.9998	5–100	14.7494	0.2638
	Liver	*y* = 28.213*x* − 115.87	*R* ^2^ = 0.9991	5–100	27.4261	0.4906
	Spleen	*y* = 38.234*x* − 66.065	*R* ^2^ = 0.9998	2–50	8.4768	0.3033
	Lung	*y* = 52.063*x* − 119.94	*R* ^2^ = 0.9991	5–100	10.7568	0.2546
	Kidney	*y* = 18.133*x* − 32.32	*R* ^2^ = 0.9993	2–50	7.7062	0.2757
	Brain	*y* = 5.7194*x* + 13.483	*R* ^2^ = 0.9994	2–50	2.3118	0.0827
	Stomach	*y* = 52.209*x* − 32.98	*R* ^2^ = 0.9991	5–250	9.2349	0.9419
	Uterus	*y* = 43.952*x* − 104.76	*R* ^2^ = 0.9993	5–50	8.5206	0.6613
	Ovary	*y* = 33.871*x* − 78.24	*R* ^2^ = 0.9993	5–100	5.5678	0.5468
	Intestine	*y* = 41.795*x* − 203.61	*R* ^2^ = 0.9991	5–100	4.2632	0.0762

Naringin	Heart	*y* = 8.8875*x* − 16.6369	*R* ^2^ = 0.9990	5–1,000	3.0936	0.0878
	Liver	*y* = 2.2546*x* − 2.937	*R* ^2^ = 0.9996	10–10,000	7.5588	0.0015
	Spleen	*y* = 2.3615*x* + 62.727	*R* ^2^ = 0.9992	5–10,000	7.5326	0.0057
	Lung	*y* = 2.7462*x* + 20.433	*R* ^2^ = 0.9997	5–10,000	1.0489	0.0839
	Kidney	*y* = 1.4648*x* + 125.32	*R* ^2^ = 0.9993	5–10,000	10.7813	0.0128
	Brain	*y* = 5.1578*x* +9.1048	*R* ^2^ = 0.9990	5–1,000	4.0361	0.0388
	Stomach	*y* = 2.4286*x* + 376.32	*R* ^2^ = 0.9993	5–20,000	14.1719	0.0236
	Uterus	*y* = 3.9995*x* + 43.448	*R* ^2^ = 0.9990	5–5,000	9.2749	0.0171
	Ovary	*y* = 1.6439*x* + 171.55	*R* ^2^ = 0.9995	5–10,000	8.4004	0.0075
	Intestine	*y* = 2.4255*x* + 344.31	*R* ^2^ = 0.9990	5–20,000	4.4006	0.0207

Eriodictyol	Liver	*y* = 25.147*x* − 94.45	*R* ^2^ = 0.9994	5–100	6.1292	0.1096
	Spleen	*y* = 9.1535*x* − 8.4711	*R* ^2^ = 0.9995	2–50	1.4348	0.0513
	Lung	*y* = 16.877*x* − 61.056	*R* ^2^ = 0.9992	5–100	7.2109	0.1289
	Stomach	*y* = 26.831*x* − 85.59	*R* ^2^ = 0.9991	5–250	10.1807	0.1236
	Uterus	*y* = 11.401*x* − 3.982	*R* ^2^ = 0.9995	2–50	5.3807	0.1925
	Ovary	*y* = 18.425*x* − 65.716	*R* ^2^ = 0.9992	5–100	4.4218	0.2579
	Intestine	*y* = 25.138*x* − 3.45	*R* ^2^ = 0.9993	5–250	4.3516	0.3611

Naringenin	Heart	*y* = 21.6837*x* + 27.6507	*R* ^2^ = 0.9996	5–250	9.4029	0.1579
	Liver	*y* = 27.289*x* + 323.82	*R* ^2^ = 0.9995	5–1,000	6.6046	0.2573
	Spleen	*y* = 25.421*x* − 23.549	*R* ^2^ = 0.9991	5–250	6.4353	0.3780
	Lung	*y* = 36.466*x* − 98.509	*R* ^2^ = 0.9992	5–250	3.8363	0.1696
	Kidney	*y* = 20.267*x* + 108.13	*R* ^2^ = 0.9990	5–250	8.1007	0.3102
	Brain	*y* = 5.1578*x* +9.1048	*R* ^2^ = 0.9990	2–50	2.5110	0.0898
	Stomach	*y* = 23.599*x* + 267.38	*R* ^2^ = 0.9996	5–1,000	6.5617	0.2572
	Uterus	*y* = 27.424*x* − 20.783	*R* ^2^ = 0.9990	5–250	1.1989	0.0976
	Ovary	*y* = 29.424*x* − 112.58	*R* ^2^ = 0.9995	5–250	1.1010	0.0822
	Intestine	*y* = 30.804*x* − 2.1048	*R* ^2^ = 0.9991	5–1,000	1.3461	0.2736

Kaempferol	Liver	*y* = 13.699*x* − 62.076	*R* ^2^ = 0.9992	5–100	9.1285	0.2192
	Spleen	*y* = 10.466*x* − 41.289	*R* ^2^ = 0.9997	10–250	2.5830	0.1024
	Lung	*y* = 7.384*x* + 18.317	*R* ^2^ = 0.9997	2–50	2.0984	0.0751
	Kidney	*y* = 3.2041*x* − 2.1837	*R* ^2^ = 0.9991	10–100	3.3257	0.0593
	Stomach	*y* = 12.132*x* + 31.51	*R* ^2^ = 0.9995	2–50	4.3045	0.1540
	Uterus	*y* = 11.729*x* − 9.8825	*R* ^2^ = 0.9990	2–50	5.8182	0.2082
	Intestine	*y* = 19.205*x* − 17.2	*R* ^2^ = 0.9992	5–500	5.4486	0.2215

**Table 3:** Interday and intraday precision and accuracy data for assays of neoeriocitrin in different tissues (*n* = 6).

Tissues	Spiked (ng/mL)	Interday			Intraday		
		Mean ± SD	Precision (RSD, %)	Relative error (RE, %)	Mean ± SD	Precision (RSD, %)	Relative error (RE, %)
Heart	8.10	7.54 ± 1.12	14.85	−6.91	7.96 ± 1.17	14.70	−1.73
	121.50	112.95 ± 15.71	13.91	−7.04	113.71 ± 16.05	14.11	−6.41
	243.00	261.48 ± 27.13	10.38	7.60	262.09 ± 29.96	11.43	7.86

Liver	12.20	10.49 ± 1.06	10.10	−14.02	10.47 ± 1.27	12.13	−14.18
	610.00	594.67 ± 14.10	2.37	−2.51	590.92 ± 16.50	2.79	−3.13
	3,660.00	3,358.79 ± 264.49	7.87	−8.23	3,397.03 ± 245.48	7.23	−7.18

Spleen	8.10	9.28 ± 0.54	5.82	14.57	9.17 ± 0.57	6.22	13.21
	810.00	803.86 ± 14.51	1.81	−0.76	811.04 ± 12.00	1.48	0.13
	4,050.00	3,809.56 ± 481.25	12.63	−5.94	3,797.20 ± 425.73	11.21	6.24

Lung	12.20	12.59 ± 1.15	9.13	3.20	12.35 ± 1.27	10.28	1.23
	1,220.00	1,056.82 ± 116.83	11.05	−13.38	1,094.38 ± 129.81	11.86	−10.30
	4,880.00	4,674.43 ± 120.35	2.57	−4.21	4,678.96 ± 145.06	3.10	−4.12

Kidney	8.10	9.27 ± 0.29	3.13	14.44	9.04 ± 0.23	2.54	11.60
	121.50	122.73 ± 17.94	14.62	1.01	118.66 ± 16.84	14.19	−2.34
	243.00	240.32 ± 35.32	14.70	−1.10	249.55 ± 37.09	14.86	2.70

Brain	8.10	8.80 ± 1.31	14.89	8.64	9.21 ± 0.89	9.66	13.70
	40.50	35.93 ± 5.23	14.56	−11.28	37.35 ± 5.10	13.65	−7.78
	81.00	87.50 ± 6.45	7.37	8.02	86.92 ± 7.89	9.08	7.31

Stomach	8.10	9.15 ± 0.89	9.73	12.96	8.38 ± 0.77	9.19	3.46
	810.00	888.08 ± 50.38	5.67	9.64	902.84 ± 41.59	4.61	11.46
	4,050.00	4,190.72 ± 365.94	8.73	3.47	4,373.61 ± 275.62	6.30	7.99

Uterus	8.10	8.86 ± 1.27	14.33	9.38	9.11 ± 1.36	14.93	12.47
	121.50	119.15 ± 15.40	12.92	−1.93	121.49 ± 13.61	11.20	−0.01
	243.00	233.53 ± 32.20	13.79	−3.90	237.59 ± 25.69	10.81	−2.23

Ovary	8.10	7.95 ± 1.03	12.96	−1.85	8.01 ± 1.02	12.73	−1.11
	121.50	109.23 ± 11.97	10.96	−10.10	115.29 ± 9.59	8.32	−5.11
	243.00	231.95 ± 30.92	13.33	−4.55	232.67 ± 30.02	12.90	−4.25

Intestine	8.10	8.76 ± 0.81	9.25	8.15	8.96 ± 0.74	8.26	10.62
	810.00	783.47 ± 54.18	6.92	−3.28	763.63 ± 29.14	3.82	-5.72
	4,050.00	4,236.54 ± 411.35	9.71	4.61	4,218.46 ± 458.40	10.87	4.16

**Table 4:** Interday and intraday precision and accuracy data for assays of luteolin-7-*O-β*-D-glucoside in different tissues (*n* = 6).

Tissues	Spiked (ng/mL)	Interday			Intraday		
		Mean ± SD	Precision (RSD, %)	Relative error (RE, %)	Mean ± SD	Precision (RSD, %)	Relative error (RE, %)
Heart	5.05	5.09 ± 0.56	11.00	0.79	5.22 ± 0.64	12.26	3.37
	10.10	9.74 ± 1.30	13.35	−3.56	10.28 ± 1.15	11.19	1.78
	25.25	23.44 ± 2.76	11.77	−7.17	22.49 ± 2.68	11.92	−10.93

Liver	5.05	4.81 ± 0.69	14.35	−4.75	4.95 ± 0.70	14.14	−1.98
	10.10	9.23 ± 0.98	10.62	−8.61	9.12 ± 1.17	12.83	−9.70
	25.25	24.52 ± 2.55	10.40	−2.89	25.12 ± 1.67	6.65	−0.51

Spleen	5.05	4.70 ± 0.62	13.19	−6.93	4.86 ± 0.70	14.40	−3.76
	10.10	10.67 ± 1.51	14.15	5.64	11.05 ± 1.45	13.12	9.41
	25.25	25.98 ± 1.62	6.24	2.89	25.97 ± 1.08	4.16	2.85

Lung	8.10	8.61 ± 0.64	7.43	6.30	8.87 ± 0.64	7.22	9.51
	40.50	39.29 ± 5.51	14.02	−2.99	36.05 ± 2.02	5.60	−10.99
	81.00	82.44 ± 3.24	3.93	1.78	82.32 ± 3.53	4.29	1.63

Kidney	5.05	4.90 ± 0.62	12.65	−2.97	4.78 ± 0.56	11.72	−5.35
	10.10	10.43 ± 1.50	14.38	3.27	10.70 ± 1.45	13.55	5.94
	25.25	25.07 ± 2.34	9.33	−0.71	24.93 ± 2.90	11.63	−1.27

Brain	5.05	4.82 ± 0.43	8.92	−4.55	4.63 ± 0.41	8.86	−8.32
	10.10	10.40 ± 1.48	14.23	2.97	10.36 ± 1.09	10.52	2.57
	25.25	24.69 ± 3.04	12.31	−2.22	25.89 ± 3.01	11.63	2.53

Stomach	8.10	7.94 ± 0.76	9.57	−1.98	7.55 ± 0.28	3.71	−6.79
	40.50	40.41 ± 4.15	10.27	−0.22	39.04 ± 2.31	5.92	−3.60
	81.00	81.58 ± 5.19	6.36	0.72	83.26 ± 5.63	6.76	2.79

Uterus	5.05	4.60 ± 0.61	13.26	−8.91	4.57 ± 0.54	11.82	−9.50
	10.10	8.99 ± 0.43	4.78	−10.99	9.08 ± 0.52	5.73	−10.10
	25.25	24.19 ± 2.98	12.32	−4.20	23.33 ± 2.55	10.93	−7.60

Ovary	5.05	4.57 ± 0.63	13.79	−9.50	4.74 ± 0.71	14.98	−6.14
	10.10	9.61 ± 1.37	14.26	−4.85	9.90 ± 1.37	13.84	−1.98
	25.25	24.48 ± 1.22	4.98	−3.05	23.78 ± 0.63	2.65	−5.82

Intestine	8.10	8.19 ± 1.02	12.45	1.11	7.63 ± 0.65	8.52	−5.80
	40.50	39.08 ± 5.23	13.38	−3.51	37.96 ± 3.89	10.25	−6.27
	81.00	82.05 ± 9.79	11.93	1.30	78.50 ± 10.40	13.25	−3.09

**Table 5:** Interday and intraday precision and accuracy data for assays of astragalin in different tissues (*n* = 6).

Tissues	Spiked (ng/mL)	Interday			Intraday		
		Mean ± SD	Precision (RSD, %)	Relative error (RE, %)	Mean ± SD	Precision (RSD, %)	Relative error (RE, %)
Heart	8.10	8.08 ± 1.07	13.24	−0.25	7.91 ± 0.93	11.76	−2.35
	40.50	38.56 ± 3.40	8.82	−4.79	37.90 ± 3.94	10.40	−6.42
	81.00	84.75 ± 8.92	10.53	4.63	83.60 ± 11.21	13.41	3.21

Liver	8.10	9.12 ± 1.20	13.16	12.59	9.08 ± 1.06	11.67	12.10
	40.50	37.17 ± 4.65	12.51	−8.22	36.06 ± 3.97	11.01	−10.96
	81.00	82.91 ± 5.22	6.30	2.36	82.43 ± 3.95	4.79	1.77

Spleen	5.05	4.55 ± 0.60	13.19	−9.90	4.61 ± 0.52	11.28	−8.71
	10.10	11.51 ± 1.47	12.77	13.96	11.42 ± 1.23	10.77	13.07
	25.25	24.75 ± 3.68	14.87	−1.98	22.94 ± 3.07	13.38	−9.15

Lung	8.10	8.78 ± 1.09	12.41	8.40	8.85 ± 1.24	14.01	9.26
	40.50	43.12 ± 4.18	9.69	6.47	42.70 ± 2.98	6.98	5.43
	81.00	85.32 ± 6.58	7.71	5.33	86.32 ± 5.75	6.66	6.57

Kidney	5.05	4.66 ± 0.64	13.73	−7.72	4.98 ± 0.51	10.24	−1.39
	10.10	10.23 ± 1.26	12.32	1.29	10.39 ± 1.23	11.84	2.87
	25.25	23.85 ± 3.52	14.76	−5.54	23.85 ± 3.21	13.46	−5.54

Brain	5.05	4.95 ± 0.72	14.55	−1.98	4.65 ± 0.61	13.12	−7.92
	10.10	9.43 ± 1.15	12.20	−6.63	8.89 ± 0.87	9.79	−11.98
	25.25	23.40 ± 2.89	12.35	−7.33	22.85 ± 2.34	10.24	−9.50

Stomach	8.10	8.89 ± 1.20	13.50	9.75	9.13 ± 1.32	14.46	12.72
	81.00	86.11 ± 8.14	9.45	6.31	87.58 ± 9.09	10.38	8.12
	162.00	154.67 ± 21.08	13.63	−4.52	165.48 ± 16.47	9.95	2.15

Uterus	5.05	4.80 ± 0.60	12.50	−4.95	4.61 ± 0.46	9.98	−8.71
	10.10	10.15 ± 1.47	14.48	0.50	9.68 ± 1.30	13.43	−4.16
	25.25	25.85 ± 3.22	12.46	2.38	25.33 ± 3.72	14.69	0.32

Ovary	8.10	9.10 ± 1.21	13.30	12.35	9.11 ± 1.22	13.39	12.47
	40.50	41.81 ± 6.20	14.83	3.23	44.33 ± 5.85	13.20	9.46
	81.00	87.39 ± 12.01	13.74	7.89	85.47 ± 11.40	13.34	5.52

Intestine	8.10	9.13 ± 1.27	13.91	12.72	8.71 ± 1.12	12.86	7.53
	40.50	38.26 ± 5.07	13.25	−5.53	40.21 ± 5.18	12.88	−0.72
	81.00	82.56 ± 8.73	10.57	1.93	78.73 ± 8.08	10.26	−2.80

**Table 6:** Interday and intraday precision and accuracy data for assays of naringin in different tissues (*n* = 6).

Tissues	Spiked (ng/mL)	Interday			Intraday		
		Mean ± SD	Precision (RSD, %)	Relative error (RE, %)	Mean ± SD	Precision (RSD, %)	Relative error (RE, %)
Heart	8.10	9.24 ± 1.38	14.94	14.07	9.16 ± 1.26	13.76	13.09
	405.00	380.94 ± 43.17	11.33	−5.94	377.25 ± 55.15	14.62	−6.85
	810.00	836.12 ± 75.60	9.04	3.22	837.23 ± 64.62	7.72	3.36

Liver	12.20	11.91 ± 1.16	9.74	−2.38	12.22 ± 1.31	10.72	0.16
	1,220.00	1,083.38 ± 128.98	11.91	−11.20	1,118.43 ± 150.05	13.42	−8.33
	4,880.00	4,989.75 ± 685.15	13.73	2.25	4,904.97 ± 645.18	13.15	0.51

Spleen	8.10	9.10 ± 1.26	13.85	12.35	8.83 ± 1.26	14.27	9.01
	810.00	825.26 ± 66.35	8.04	1.88	854.30 ± 62.59	7.33	5.47
	4,050.00	4,004.43 ± 587.31	14.67	−1.12	4,178.48 ± 568.58	13.61	3.17

Lung	8.10	9.21 ± 0.84	9.12	13.70	9.15 ± 0.83	9.07	12.96
	810.00	845.41 ± 101.35	11.99	4.37	839.70 ± 119.76	14.26	3.67
	4,050.00	4,011.38 ± 587.19	14.64	−0.95	4,247.34 ± 556.90	13.11	4.87

Kidney	8.10	8.98 ± 1.06	11.80	10.86	8.69 ± 0.54	6.21	7.28
	810.00	863.67 ± 90.41	10.47	6.63	878.63 ± 109.04	12.41	8.47
	4,050.00	3,956.27 ± 574.77	14.53	−2.31	4,029.43 ± 591.13	14.67	−0.51

Brain	8.10	8.13 ± 1.08	13.28	0.37	8.53 ± 1.14	13.36	5.31
	405.00	415.00 ± 51.43	12.39	2.47	399.72 ± 52.49	13.13	−1.30
	810.00	849.97 ± 118.72	13.97	4.93	889.89 ± 130.79	14.70	9.86

Stomach	8.10	8.92 ± 1.22	13.68	10.12	9.03 ± 1.07	11.85	11.48
	810.00	783.57 ± 92.13	11.76	−3.26	754.08 ± 70.59	9.36	−6.90
	8,100.00	7,802.03 ± 571.75	7.33	−3.68	7,806.89 ± 735.92	9.43	−3.62

Uterus	8.10	9.02 ± 0.89	9.87	11.36	9.22 ± 0.73	7.92	13.83
	1,215.00	1,257.33 ± 168.44	13.40	3.48	1,233.89 ± 179.47	14.55	1.55
	2,430.00	2,361.88 ± 303.00	12.83	−2.80	2,311.52 ± 291.80	12.62	−4.88

Ovary	8.10	8.63 ± 1.19	13.79	6.54	9.12 ± 1.06	11.62	12.59
	810.00	857.76 ± 56.18	6.55	5.90	869.99 ± 58.59	6.73	7.41
	4,050.00	4,025.32 ± 476.37	11.83	−0.61	4,004.08 ± 498.00	12.44	−1.13

Intestine	8.10	8.74 ± 1.28	14.65	7.90	8.73 ± 1.21	13.86	7.78
	810.00	848.01 ± 108.63	12.81	4.69	818.73 ± 113.46	13.86	1.08
	8,100.00	8,290.57 ± 1,207.41	14.56	2.35	8,888.41 ± 968.47	10.90	9.73

**Table 7:** Interday and intraday precision and accuracy data for assays of eriodictyol in different tissues (*n* = 6).

Tissues	Spiked (ng/mL)	Interday			Intraday		
		Mean ± SD	Precision (RSD, %)	Relative error (RE, %)	Mean ± SD	Precision (RSD, %)	Relative error (RE, %)
Liver	8.10	8.90 ± 1.28	14.38	9.88	8.58 ± 1.25	14.57	5.93
	40.50	38.20 ± 4.04	10.58	−5.68	36.81 ± 3.60	9.78	−9.11
	81.00	84.30 ± 10.68	12.67	4.07	86.45 ± 11.11	12.85	6.73

Spleen	5.05	4.90 ± 0.55	11.22	−2.97	4.75 ± 0.65	13.68	−5.94
	10.10	11.50 ± 0.98	8.52	13.86	11.61 ± 1.12	9.65	14.95
	25.25	22.66 ± 2.81	12.40	−10.26	22.78 ± 2.62	11.50	−9.78

Lung	8.10	8.59 ± 0.90	10.48	6.05	9.11 ± 0.49	5.38	12.47
	40.50	41.34 ± 5.82	14.08	2.07	43.80 ± 5.67	12.95	8.15
	81.00	81.34 ± 10.47	12.87	0.42	78.13 ± 9.28	11.88	−3.54

Stomach	8.10	9.14 ± 1.31	14.33	12.84	9.21 ± 1.18	12.81	13.70
	81.00	81.91 ± 7.07	8.63	1.12	80.93 ± 7.10	8.77	−0.09
	162.00	159.83 ± 23.14	14.48	−1.34	166.06 ± 24.72	14.89	2.51

Uterus	5.05	5.24 ± 0.74	14.12	3.76	5.43 ± 0.63	11.60	7.52
	10.10	10.16 ± 1.38	13.58	0.59	10.34 ± 1.39	13.44	2.38
	25.25	22.68 ± 2.90	12.79	−10.18	22.85 ± 3.59	13.09	−9.50

Ovary	8.10	8.91 ± 1.06	11.90	10.00	8.55 ± 1.08	12.63	5.56
	40.50	38.38 ± 5.30	13.81	−5.23	39.78 ± 4.77	11.99	−1.78
	81.00	87.31 ± 7.86	9.00	7.79	91.95 ± 4.08	4.44	13.52

Intestine	8.10	8.83 ± 1.18	13.36	9.01	8.92 ± 1.01	11.32	10.12
	81.00	80.17 ± 9.98	12.45	−1.02	80.27 ± 10.22	12.73	−0.90
	162.00	175.52 ± 20.24	11.53	8.35	176.33 ± 21.61	12.26	8.85

**Table 8:** Interday and intraday precision and accuracy data for assays of naringenin in different tissues (*n* = 6).

Tissues	Spiked (ng/mL)	Interday			Intraday		
		Mean ± SD	Precision (RSD, %)	Relative error (RE, %)	Mean ± SD	Precision (RSD, %)	Relative error (RE, %)
Heart	8.10	8.13 ± 1.13	13.90	0.37	8.16 ± 1.08	13.24	0.74
	81.00	82.92 ± 8.68	10.47	2.37	86.88 ± 6.85	7.88	7.26
	162.00	160.32 ± 22.94	14.31	−1.04	167.09 ± 24.86	14.88	3.14

Liver	8.10	8.63 ± 1.20	13.90	6.54	9.09 ± 1.24	13.64	12.22
	405.00	427.60 ± 51.68	12.09	5.58	453.02 ± 42.58	9.40	11.86
	810.00	775.22 ± 90.48	11.67	−4.29	817.22 ± 80.89	9.90	0.89

Spleen	8.10	9.24 ± 1.01	10.93	14.07	9.13 ± 0.93	10.19	12.72
	81.00	85.43 ± 6.78	7.94	5.47	86.86 ± 4.52	5.20	7.23
	162.00	152.55 ± 18.72	12.27	−5.83	151.26 ± 21.45	14.18	−6.63

Lung	8.10	8.81 ± 1.26	14.30	8.77	8.69 ± 0.54	6.21	7.28
	81.00	79.67 ± 9.12	11.45	−1.64	83.38 ± 8.76	10.51	2.94
	162.00	155.57 ± 22.84	14.68	−3.97	151.47 ± 19.85	13.10	−6.50

Kidney	8.10	8.43 ± 1.12	13.29	4.07	8.70 ± 1.21	13.91	7.41
	81.00	82.07 ± 8.79	10.71	1.32	85.59 ± 7.93	9.27	5.67
	162.00	163.46 ± 24.17	14.79	0.90	177.97 ± 10.54	5.92	9.86

Brain	5.05	4.71 ± 0.64	13.59	−6.73	4.76 ± 0.71	14.92	−5.74
	10.10	11.12 ± 1.54	13.85	10.10	11.00 ± 1.54	14.00	8.91
	25.25	26.41 ± 3.44	13.03	4.59	26.19 ± 3.45	13.17	3.72

Stomach	8.10	8.99 ± 1.19	13.24	10.99	8.91 ± 0.81	9.09	10.00
	405.00	412.81 ± 58.73	14.23	1.93	441.06 ± 49.05	11.12	8.90
	810.00	833.38 ± 105.06	12.61	2.89	818.43 ± 80.14	9.79	1.04

Uterus	8.10	8.57 ± 1.13	13.19	5.80	8.96 ± 1.06	11.83	10.62
	81.00	79.55 ± 10.82	13.60	−1.79	80.82 ± 11.94	14.77	−0.22
	162.00	163.07 ± 24.08	14.77	0.66	171.73 ± 24.88	14.49	6.01

Ovary	8.10	8.29 ± 1.08	13.03	2.35	8.66 ± 1.18	13.63	6.91
	81.00	82.27 ± 10.04	12.20	1.57	84.42 ± 12.23	14.49	4.22
	162.00	163.78 ± 23.19	14.16	1.10	164.51 ± 17.65	10.73	1.55

Intestine	8.10	7.63 ± 0.85	11.14	−5.80	7.78 ± 1.05	13.50	−3.95
	405.00	404.43 ± 55.41	13.70	−0.14	428.48 ± 52.94	12.36	5.80
	810.00	770.23 ± 82.03	10.65	−4.91	784.08 ± 102.02	13.01	−3.20

**Table 9:** Interday and intraday precision and accuracy data for assays of kaempferol in different tissues (*n* = 6).

Tissues	Spiked (ng/mL)	Interday			Intraday		
		Mean ± SD	Precision (RSD, %)	Relative error (RE, %)	Mean ± SD	Precision (RSD, %)	Relative error (RE, %)
Liver	8.10	8.50 ± 1.23	14.47	4.94	8.36 ± 1.19	14.23	3.21
	40.50	45.97 ± 6.78	14.75	13.51	45.61 ± 5.04	11.05	12.62
	81.00	82.90 ± 11.10	13.39	2.35	85.23 ± 12.01	14.09	5.22

Spleen	12.20	11.06 ± 1.54	13.92	−9.34	10.75 ± 0.72	6.70	−11.89
	91.50	97.07 ± 12.65	13.03	6.09	94.94 ± 10.93	11.51	3.76
	183.00	188.84 ± 25.77	13.65	3.19	180.04 ± 22.90	12.72	−1.62

Lung	5.05	5.08 ± 0.63	12.40	0.59	4.76 ± 0.30	6.30	−5.74
	10.10	9.34 ± 1.28	13.70	−7.52	8.67 ± 0.50	5.77	−14.16
	25.25	23.74 ± 3.26	13.73	−5.98	22.77 ± 3.05	13.39	−9.82

Kidney	12.20	10.71 ± 1.12	10.46	−12.21	11.37 ± 0.55	4.84	−6.80
	42.70	41.36 ± 4.67	11.29	−3.14	43.56 ± 4.11	9.44	2.01
	85.40	86.37 ± 8.43	9.76	1.14	90.48 ± 6.45	7.13	5.95

Stomach	5.05	4.81 ± 0.69	14.35	−4.75	4.82 ± 0.59	12.24	−4.55
	10.10	10.51 ± 1.41	13.42	4.06	10.64 ± 1.47	13.82	5.35
	25.25	24.14 ± 3.37	13.96	−4.40	25.50 ± 3.41	13.37	0.99

Uterus	5.05	4.89 ± 0.54	11.04	−3.17	4.91 ± 0.50	10.18	−2.77
	10.10	10.35 ± 1.41	13.62	2.48	10.16 ± 1.10	10.83	0.59
	25.25	24.16 ± 2.84	11.75	−4.32	23.47 ± 3.31	14.10	−7.05

Intestine	8.10	8.31 ± 1.04	12.52	2.59	8.32 ± 1.15	13.82	2.72
	121.50	105.10 ± 11.92	11.34	−13.50	104.54 ± 5.05	4.83	−13.96
	243.00	264.29 ± 39.44	14.92	8.76	271.43 ± 36.43	13.42	11.70

**Table 10:** Recovery and matrix effect study of neoeriocitrin in rat tissues (*n* = 6).

Tissues	Spiked (ng/mL)	Recovery		Matrix effects	
		Mean (%) ± SD	RSD (%)	Mean (%) ± SD	RSD (%)
Heart	8.10	94.72 ± 14.10	14.89	95.70 ± 13.74	14.36
	121.50	91.22 ± 13.65	14.96	95.21 ± 13.09	13.75
	243.00	105.88 ± 11.56	10.92	109.53 ± 11.31	10.33

Liver	12.20	86.98 ± 9.39	10.80	84.95 ± 9.27	10.91
	610.00	97.31 ± 2.54	2.61	97.17 ± 2.44	2.51
	3,660.00	90.68 ± 7.51	8.28	93.69 ± 6.13	6.54

Spleen	8.10	115.23 ± 7.17	6.22	115.8 ± 6.58	5.68
	810.00	99.6 ± 1.74	1.75	99.59 ± 1.76	1.77
	4,050.00	91.21 ± 10.74	11.78	96.67 ± 11.20	11.59

Lung	12.20	103.66 ± 10.52	10.15	101.25 ± 9.04	8.93
	1,220.00	87.54 ± 10.41	11.89	88.18 ± 9.83	11.15
	4,880.00	95.55 ± 2.68	2.80	96.10 ± 2.62	2.73

Kidney	8.10	114.05 ± 3.81	3.34	115.65 ± 2.59	2.24
	121.50	100.88 ± 14.50	14.37	98.47 ± 14.56	14.79
	243.00	98.5 ± 14.22	14.44	102.34 ± 13.24	12.94

Brain	8.10	111.41 ± 14.50	13.01	112.94 ± 13.84	12.25
	40.50	90.48 ± 13.11	14.49	89.76 ± 13.16	14.66
	81.00	107.02 ± 8.46	7.91	108.47 ± 8.82	8.13

Stomach	8.10	112 ± 12.00	10.71	116.4 ± 8.33	7.16
	810.00	109.15 ± 6.82	6.25	111.59 ± 4.46	4.00
	4,050.00	105.59 ± 7.97	7.55	104.97 ± 8.96	8.54

Uterus	8.10	108.3 ± 14.27	13.18	113.04 ± 14.59	12.91
	121.50	101.6 ± 10.34	10.18	96.07 ± 13.08	13.62
	243.00	97.02 ± 14.20	14.64	96.53 ± 14.07	14.58

Ovary	8.10	98.12 ± 14.25	14.52	98.86 ± 14.13	14.29
	121.50	91.95 ± 9.48	10.31	91.85 ± 9.65	10.51
	243.00	92.92 ± 12.43	13.38	98.22 ± 12.04	12.26

Intestine	8.10	107.31 ± 10.98	10.23	111.09 ± 7.96	7.17
	810.00	97.25 ± 7.34	7.55	94.24 ± 3.12	3.31
	4,050.00	106.03 ± 10.66	10.05	102.82 ± 10.25	9.97

**Table 11:** Recovery and matrix effect study of luteolin-7-*O-β*-D-glucoside in rat tissues (*n* = 6).

Tissues	Spiked (ng/mL)	Recovery		Matrix effects	
		Mean (%) ± SD	RSD (%)	Mean (%) ± SD	RSD (%)
Heart	5.05	100.87 ± 12.35	12.24	102.97 ± 11.06	10.74
	10.10	99.96 ± 10.70	10.70	97.19 ± 14.26	14.67
	25.25	90.02 ± 9.44	10.49	92.67 ± 12.21	13.18

Liver	5.05	94.93 ± 14.20	14.96	97.94 ± 13.43	13.71
	10.10	92.00 ± 10.76	11.70	89.94 ± 10.10	11.23
	25.25	95.58 ± 10.45	10.93	100.58 ± 6.20	6.16

Spleen	5.05	93.39 ± 13.76	14.73	95.60 ± 12.14	12.70
	10.10	106.57 ± 14.55	13.65	107.84 ± 14.56	13.50
	25.25	104.63 ± 5.39	5.15	101.16 ± 5.34	5.28

Lung	8.10	107.38 ± 8.33	7.76	107.83 ± 7.81	7.24
	40.50	95.09 ± 12.26	12.79	92.55 ± 9.01	9.74
	81.00	101.05 ± 3.99	3.95	102.4 ± 4.14	4.04

Kidney	5.05	96.75 ± 13.77	14.23	95.49 ± 13.13	13.75
	10.10	102.34 ± 14.32	13.99	106.46 ± 14.21	13.35
	25.25	98.30 ± 10.01	10.18	99.87 ± 10.25	10.26

Brain	5.05	93.82 ± 8.47	9.03	94.06 ± 8.78	9.33
	10.10	106.65 ± 13.04	12.23	99.03 ± 12.31	12.43
	25.25	100.51 ± 11.27	11.21	98.89 ± 13.15	13.30

Stomach	8.10	94.52 ± 4.07	4.31	97.78 ± 10.50	10.74
	40.50	100.84 ± 11.08	10.99	96.03 ± 5.00	5.21
	81.00	101.98 ± 6.28	6.16	101.12 ± 7.08	7.00

Uterus	5.05	88.08 ± 10.75	12.20	93.66 ± 11.69	12.48
	10.10	89.52 ± 4.55	5.08	89.29 ± 4.69	5.25
	25.25	92.2 ± 8.76	9.50	96.70 ± 12.98	13.42

Ovary	5.05	91.09 ± 12.84	14.10	92.83 ± 12.48	13.44
	10.10	97.72 ± 13.49	13.80	94.93 ± 14.17	14.93
	25.25	95.52 ± 3.65	3.82	96.21 ± 4.98	5.18

Intestine	8.10	97.78 ± 10.52	10.76	99.04 ± 12.78	12.90
	40.50	98.43 ± 13.42	13.63	92.34 ± 8.89	9.63
	81.00	99.19 ± 12.22	12.32	99.90 ± 12.97	12.98

**Table 12:** Recovery and matrix effect study of astragalin in rat tissues (*n* = 6).

Tissues	Spiked (ng/mL)	Recovery		Matrix effects	
		Mean (%) ± SD	RSD (%)	Mean (%) ± SD	RSD (%)
Heart	8.10	96.05 ± 10.66	11.10	101.93 ± 13.66	13.40
	40.50	93.71 ± 8.42	8.99	95.41 ± 9.36	9.81
	81.00	104.43 ± 12.29	11.77	103.7 ± 12.04	11.61

Liver	8.10	115.16 ± 14.23	12.36	119.16 ± 14.67	12.31
	40.50	88.31 ± 8.64	9.78	93.07 ± 12.34	13.26
	81.00	100.5 ± 5.09	5.06	103.75 ± 6.12	5.90

Spleen	5.05	93.31 ± 9.96	10.67	87.88 ± 11.81	13.44
	10.10	118.57 ± 10.60	8.94	112.75 ± 14.93	13.24
	25.25	94.99 ± 14.01	14.75	95.34 ± 13.54	14.20

Lung	8.10	109.23 ± 14.92	13.66	108.4 ± 14.06	12.97
	40.50	103.39 ± 7.85	7.59	108.74 ± 9.74	8.96
	81.00	107.62 ± 6.59	6.12	104.04 ± 8.35	8.03

Kidney	5.05	94.5 ± 12.84	13.59	95.33 ± 11.54	12.11
	10.10	99.5 ± 12.95	13.02	104.44 ± 11.11	10.64
	25.25	93.96 ± 13.12	13.96	94.98 ± 13.52	14.23

Brain	5.05	93.98 ± 11.22	11.94	97.5 ± 13.92	14.28
	10.10	89.98 ± 8.59	9.55	92.55 ± 12.47	13.47
	25.25	89.2 ± 8.53	9.56	94.42 ± 11.88	12.58

Stomach	8.10	112.99 ± 14.09	12.47	108.96 ± 14.43	13.24
	81.00	108.37 ± 9.73	8.98	105.71 ± 11.12	10.52
	162.00	97.94 ± 12.88	13.15	98.35 ± 12.24	12.45

Uterus	5.05	91.17 ± 7.91	8.68	96.04 ± 13.06	13.60
	10.10	96.22 ± 11.18	11.62	101.13 ± 14.18	14.02
	25.25	100.08 ± 12.77	12.76	103.07 ± 14.14	13.72

Ovary	8.10	108.74 ± 13.50	12.41	116.05 ± 13.30	11.46
	40.50	104.44 ± 14.80	14.17	107.02 ± 13.64	12.75
	81.00	109.00 ± 14.30	13.12	104.89 ± 14.40	13.73

Intestine	8.10	109.56 ± 14.08	12.85	111.85 ± 14.31	12.79
	40.50	95.85 ± 13.47	14.05	96.94 ± 12.25	12.64
	81.00	99.52 ± 10.07	10.12	100.56 ± 11.45	11.39

**Table 13:** Recovery and matrix effect study of naringin in rat tissues (*n* = 6).

Tissues	Spiked (ng/mL)	Recovery		Matrix effects	
		Mean (%) ± SD	RSD (%)	Mean (%) ± SD	RSD (%)
Heart	8.10	110.00 ± 14.19	12.90	117.51 ± 14.62	12.44
	405.00	93.51 ± 11.82	12.64	93.89 ± 11.91	12.69
	810.00	101.07 ± 8.60	8.51	105.49 ± 8.39	7.95

Liver	12.20	97.87 ± 10.65	10.88	99.51 ± 9.41	9.46
	1,220.00	89.61 ± 11.61	12.96	90.30 ± 11.09	12.28
	4,880.00	99.40 ± 13.63	13.71	103.70 ± 14.18	13.67

Spleen	8.10	109.60 ± 14.70	13.41	112.52 ± 14.42	12.82
	810.00	103.36 ± 8.21	7.94	103.27 ± 8.33	8.07
	4,050.00	101.09 ± 14.04	13.89	100.10 ± 14.86	14.85

Lung	8.10	115.98 ± 9.88	8.52	114.91 ± 11.15	9.70
	810.00	103.72 ± 13.87	13.37	104.46 ± 13.99	13.39
	4,050.00	100.14 ± 13.93	13.91	102.61 ± 12.94	12.61

Kidney	8.10	112.86 ± 13.55	12.01	106.12 ± 6.39	6.02
	810.00	108.24 ± 11.67	10.78	106.49 ± 12.47	11.71
	4,050.00	96.03 ± 14.21	14.80	100.79 ± 13.41	13.30

Brain	8.10	102.10 ± 14.19	13.90	102.74 ± 13.52	13.16
	405.00	102.58 ± 14.19	13.83	99.34 ± 11.32	11.40
	810.00	106.80 ± 14.57	13.64	107.01 ± 14.37	13.43

Stomach	8.10	107.26 ± 14.80	13.80	114.20 ± 12.91	11.30
	810.00	97.56 ± 12.51	12.82	93.00 ± 7.55	8.12
	8,100.00	96.18 ± 7.88	8.19	96.52 ± 7.87	8.15

Uterus	8.10	114.62 ± 8.67	7.56	112.15 ± 12.13	10.82
	1,215.00	101.9 ± 14.88	14.60	103.53 ± 14.50	14.01
	2,430.00	98.79 ± 13.24	13.40	93.95 ± 10.73	11.42

Ovary	8.10	107.31 ± 13.35	12.44	110.67 ± 12.10	10.93
	810.00	105.44 ± 7.65	7.26	107.56 ± 6.27	5.83
	4,050.00	101.35 ± 12.01	11.85	97.01 ± 11.43	11.78

Intestine	8.10	104.91 ± 14.63	13.95	110.86 ± 14.79	13.34
	810.00	102.29 ± 13.48	13.18	104.20 ± 13.93	13.37
	8,100.00	106.06 ± 13.22	12.46	104.55 ± 14.54	13.91

**Table 14:** Recovery and matrix effect study of eriodictyol in rat tissues (*n* = 6).

Tissues	Spiked (ng/mL)	Recovery		Matrix effects	
		Mean (%) ± SD	RSD (%)	Mean (%) ± SD	RSD (%)
Liver	8.10	106.86 ± 14.59	13.65	109.83 ± 14.69	13.38
	40.50	91.41 ± 7.79	8.52	94.49 ± 11.13	11.78
	81.00	105.18 ± 14.43	13.72	105.09 ± 14.48	13.78

Spleen	5.05	95.80 ± 11.70	12.21	96.04 ± 11.87	12.36
	10.10	113.03 ± 10.54	9.32	115.70 ± 9.70	8.38
	25.25	92.38 ± 10.17	11.01	87.52 ± 10.86	12.41

Lung	8.10	109.31 ± 8.93	8.17	108.1 ± 11.23	10.39
	40.50	104.59 ± 14.51	13.87	104.43 ± 14.71	14.09
	81.00	96.59 ± 9.92	10.27	101.09 ± 14.34	14.19

Stomach	8.10	110.96 ± 14.26	12.85	117.43 ± 12.96	11.04
	81.00	99.02 ± 7.86	7.94	102.27 ± 9.24	9.03
	162.00	100.60 ± 14.07	13.99	99.81 ± 14.66	14.69

Uterus	5.05	102.46 ± 14.89	14.53	108.36 ± 10.90	10.06
	10.10	103.13 ± 13.69	13.27	99.58 ± 11.98	12.03
	25.25	89.11 ± 12.68	14.23	91.09 ± 12.37	13.58

Ovary	8.10	106.91 ± 11.88	11.11	109.58 ± 14.54	13.27
	40.50	96.92 ± 13.41	13.84	95.40 ± 11.35	11.90
	81.00	110.09 ± 8.83	8.02	110.08 ± 8.87	8.06

Intestine	8.10	112.94 ± 12.47	11.04	106.15 ± 14.11	13.29
	81.00	96.66 ± 12.21	12.63	101.40 ± 12.07	11.90
	162.00	106.34 ± 12.84	12.07	110.76 ± 12.31	11.11

**Table 15:** Recovery and matrix effect study of naringenin in rat tissues (*n* = 6).

Tissues	Spiked (ng/mL)	Recovery		Matrix effects	
		Mean (%) ± SD	RSD (%)	Mean (%) ± SD	RSD (%)
Heart	8.10	99.16 ± 14.17	14.29	102.00 ± 14.02	13.75
	81.00	103.12 ± 11.80	11.44	105.54 ± 8.27	7.84
	162.00	99.88 ± 14.63	14.65	101.40 ± 14.36	14.16

Liver	8.10	108.35 ± 14.84	13.70	109.38 ± 14.68	13.42
	405.00	108.53 ± 11.75	10.83	107.65 ± 13.09	12.16
	810.00	97.99 ± 10.82	11.04	97.58 ± 11.39	11.67

Spleen	8.10	110.94 ± 10.73	9.67	116.25 ± 12.71	10.93
	81.00	104.08 ± 8.55	8.21	108.27 ± 5.36	4.95
	162.00	92.22 ± 11.76	12.75	95.49 ± 12.40	12.99

Lung	8.10	112.86 ± 13.55	12.01	103.65 ± 10.11	9.75
	81.00	99.40 ± 12.26	12.33	100.99 ± 10.33	10.23
	162.00	98.08 ± 14.04	14.31	91.96 ± 11.15	12.12

Kidney	8.10	104.57 ± 14.45	13.82	106.32 ± 14.62	13.75
	81.00	101.85 ± 12.04	11.82	104.27 ± 9.04	8.67
	162.00	105.15 ± 11.94	11.36	103.82 ± 14.64	14.10

Brain	5.05	95.80 ± 12.56	13.11	91.80 ± 13.49	14.69
	10.10	108.75 ± 14.63	13.45	110.61 ± 14.02	12.68
	25.25	101.59 ± 12.78	12.58	106.93 ± 13.84	12.94

Stomach	8.10	106.79 ± 11.36	10.64	114.59 ± 13.26	11.57
	405.00	103.98 ± 14.21	13.67	105.46 ± 13.01	12.34
	810.00	105.33 ± 12.86	12.21	98.97 ± 9.74	9.84

Uterus	8.10	109.68 ± 11.56	10.54	105.90 ± 14.62	13.81
	81.00	96.79 ± 14.42	14.90	100.89 ± 13.01	12.90
	162.00	101.76 ± 14.35	14.10	103.84 ± 14.15	13.63

Ovary	8.10	104.12 ± 14.06	13.50	104.27 ± 13.92	13.35
	81.00	102.55 ± 13.60	13.26	102.73 ± 13.50	13.14
	162.00	97.63 ± 12.88	13.19	104.93 ± 12.09	11.52

Intestine	8.10	94.69 ± 11.62	12.27	95.33 ± 11.33	11.89
	405.00	102.26 ± 13.81	13.50	102.21 ± 13.87	13.57
	810.00	95.59 ± 11.24	11.76	95.96 ± 11.07	11.54

**Table 16:** Recovery and matrix effect study of kaempferol in rat tissues (*n* = 6).

Tissues	Spiked (ng/mL)	Recovery		Matrix effects	
		Mean (%) ± SD	RSD (%)	Mean (%) ± SD	RSD (%)
Liver	8.10	101.65 ± 14.31	14.08	106.96 ± 14.12	13.20
	40.50	115.4 ± 14.20	12.31	121.79 ± 10.89	8.94
	81.00	102.17 ± 14.31	14.01	104.84 ± 13.72	13.09

Spleen	12.20	93.16 ± 12.41	13.32	86.15 ± 6.77	7.86
	91.50	102.12 ± 10.97	10.74	108.20 ± 14.34	13.25
	183.00	103.48 ± 14.72	14.22	99.06 ± 10.94	11.04

Lung	5.05	100.12 ± 13.76	13.74	96.20 ± 6.49	6.75
	10.10	87.78 ± 6.08	6.93	91.86 ± 13.12	14.28
	25.25	94.43 ± 13.83	14.65	90.60 ± 12.32	13.60

Kidney	12.20	89.61 ± 9.00	10.04	90.36 ± 7.52	8.32
	42.70	99.04 ± 10.66	10.76	98.81 ± 11.00	11.13
	85.40	103.94 ± 7.94	7.64	102.19 ± 10.66	10.43

Stomach	5.05	95.6 ± 14.05	14.70	95.29 ± 14.16	14.86
	10.10	105.45 ± 14.18	13.45	103.78 ± 14.59	14.06
	25.25	97.72 ± 13.78	14.10	97.81 ± 13.67	13.98

Uterus	5.05	94.69 ± 10.39	10.97	99.45 ± 9.78	9.83
	10.10	105.13 ± 13.83	13.16	98.36 ± 10.66	10.84
	25.25	95.33 ± 12.51	13.12	93.90 ± 11.54	12.29

Intestine	8.10	104.22 ± 13.70	13.15	101.14 ± 13.81	13.65
	121.50	82.72 ± 3.60	4.35	87.29 ± 10.75	12.32
	243.00	110.63 ± 14.41	13.03	112.54 ± 14.91	13.25

**Table 17:** Stability of neoeriocitrin in rat tissues (*n* = 6).

Tissues	Spiked (ng/mL)	Room temperature			Freeze-thaw (−20°C, 3 times)			Long term (−20°C, 30 days)		
		Mean ± SD	RSD (%)	RE (%)	Mean ± SD	RSD (%)	RE (%)	Mean ± SD	RSD (%)	RE (%)
Heart	8.10	6.95 ± 0.80	11.51	−14.20	8.15 ± 1.18	14.48	0.62	7.07 ± 1.02	14.43	−12.72
	121.50	106.50 ± 6.67	6.26	−12.35	119.40 ± 16.17	13.54	−1.73	111.16 ± 5.83	5.24	−8.51
	243.00	254.81 ± 35.08	13.77	4.86	268.16 ± 21.81	8.13	10.35	249.44 ± 25.84	10.36	2.65

Liver	12.20	10.71 ± 0.95	8.87	−12.21	10.78 ± 1.30	12.06	−11.64	10.90 ± 1.52	13.94	−10.66
	610.00	596.81 ± 18.63	3.12	−2.16	592.53 ± 11.67	1.97	−2.86	586.32 ± 15.63	2.67	−3.88
	3,660.00	3,403.71 ± 343.46	10.09	−7.00	3,313.88 ± 225.54	6.81	−9.46	3,294.94 ± 300.05	9.11	−9.97

Spleen	8.10	9.30 ± 0.68	7.31	14.81	9.23 ± 0.50	5.42	13.95	9.23 ± 0.52	5.63	13.95
	810.00	809.27 ± 16.98	2.10	−0.09	798.45 ± 12.24	1.53	−1.43	806.44 ± 14.65	1.82	−0.44
	4,050.00	3,696.74 ± 532.72	14.41	−8.72	3,922.39 ± 506.99	12.93	−3.15	3,596.82 ± 365.71	10.17	11.19

Lung	12.20	13.51 ± 0.65	4.81	10.74	11.68 ± 0.63	5.39	−4.26	12.56 ± 1.38	10.99	2.95
	1,220.00	1,049.18 ± 151.19	14.41	−14.00	1,064.46 ± 105.31	9.89	−12.75	1,064.00 ± 140.09	13.17	−12.79
	4,880.00	4,693.51 ± 174.71	3.72	−3.82	4,655.35 ± 67.79	1.46	−4.60	4,706.89 ± 163.70	3.48	−3.55

Kidney	8.10	9.27 ± 0.41	4.42	14.44	9.29 ± 0.22	2.37	14.69	9.17 ± 0.31	3.38	13.21
	121.50	130.10 ± 17.12	13.16	7.08	115.37 ± 7.09	6.15	−5.05	117.29 ± 16.56	14.12	−3.47
	243.00	211.85 ± 16.00	7.55	−12.82	268.79 ± 20.77	7.73	10.61	227.65 ± 32.07	14.09	−6.32

Brain	8.10	9.10 ± 0.68	7.47	12.35	8.51 ± 0.98	11.52	5.06	8.51 ± 0.96	11.28	5.06
	40.50	36.84 ± 4.11	11.16	−9.04	35.02 ± 5.17	14.76	−13.53	35.89 ± 4.53	12.62	−11.38
	81.00	91.02 ± 4.71	5.17	12.37	83.99 ± 6.69	7.97	3.69	86.52 ± 7.86	9.08	6.81

Stomach	8.10	8.79 ± 1.10	12.51	8.52	8.03 ± 0.61	7.60	−0.86	8.42 ± 0.55	6.53	3.95
	810.00	849.82 ± 40.54	4.77	4.92	926.35 ± 17.57	1.90	14.36	867.42 ± 68.59	7.91	7.09
	4,050.00	4,342.23 ± 411.23	9.47	7.22	4,039.22 ± 283.82	7.03	−0.27	4,301.44 ± 400.81	9.32	6.21

Uterus	8.10	8.51 ± 1.02	11.99	5.06	9.22 ± 0.82	8.89	13.83	8.14 ± 1.14	14.00	0.49
	121.50	119.97 ± 12.44	10.37	−1.26	118.34 ± 15.89	13.43	−2.60	125.77 ± 17.07	13.57	3.51
	243.00	255.57 ± 29.61	11.59	5.17	211.49 ± 16.06	7.59	−12.97	232.87 ± 16.56	7.11	−4.17

Ovary	8.10	8.06 ± 0.50	6.20	−0.49	7.84 ± 0.84	10.71	−3.21	7.27 ± 0.90	12.38	−10.25
	121.50	111.60 ± 15.68	14.05	−8.15	106.87 ± 9.78	9.15	−12.04	111.12 ± 15.83	14.25	−8.54
	243.00	230.40 ± 30.17	13.09	−5.19	233.50 ± 27.75	11.88	−3.91	207.73 ± 9.56	4.60	−14.51

Intestine	8.10	8.48 ± 1.10	12.97	4.69	9.05 ± 0.45	4.97	11.73	8.62 ± 1.06	12.30	6.42
	810.00	794.00 ± 83.11	10.47	−1.98	772.96 ± 9.97	1.29	−4.57	812.09 ± 62.32	7.67	0.26
	4,050.00	4,361.18 ± 410.40	9.41	7.68	4,111.91 ± 456.07	11.09	1.53	4,607.89 ± 16.92	0.37	13.78

**Table 18:** Stability of luteolin-7-*O-β*-D-glucoside in rat tissues (*n* = 6).

Tissues	Spiked (ng/mL)	Room temperature			Freeze-thaw (−20°C, 3 times)			Long term (−20°C, 30 days)		
		Mean ± SD	RSD (%)	RE (%)	Mean ± SD	RSD (%)	RE (%)	Mean ± SD	RSD (%)	RE (%)
Heart	5.05	5.13 ± 0.66	12.87	1.58	5.07 ± 0.59	11.64	0.40	5.05 ± 0.53	10.52	−0.20
	10.10	9.36 ± 0.42	4.49	−7.33	10.12 ± 1.09	10.77	0.20	10.00 ± 0.78	7.80	−0.99
	25.25	22.45 ± 2.22	9.89	−11.09	25.44 ± 1.48	5.82	0.75	23.44 ± 1.91	8.15	−7.17

Liver	5.05	4.56 ± 0.61	13.38	−9.70	5.07 ± 0.25	4.93	0.40	5.06 ± 0.56	11.07	0.20
	10.10	8.77 ± 1.12	12.77	−13.17	9.70 ± 0.72	7.42	−3.96	9.64 ± 0.95	9.85	−4.55
	25.25	22.84 ± 1.38	6.04	−9.54	26.21 ± 1.43	5.46	3.80	23.21 ± 2.62	11.29	−8.08

Spleen	5.05	4.37 ± 0.52	11.90	−13.47	5.04 ± 0.33	6.55	−0.20	4.82 ± 0.62	12.86	−4.55
	10.10	9.78 ± 1.38	14.11	−3.17	11.58 ± 1.17	10.10	14.65	10.16 ± 1.02	10.04	0.59
	25.25	27.07 ± 1.16	4.29	7.21	24.90 ± 1.29	5.18	−1.39	26.63 ± 1.87	7.02	5.47

Lung	8.10	8.82 ± 0.74	8.39	8.89	8.41 ± 0.59	7.02	3.83	8.48 ± 0.85	10.02	4.69
	40.50	39.56 ± 5.82	14.71	−2.32	39.03 ± 3.82	9.79	−3.63	39.16± 5.77	14.73	−3.31
	81.00	82.50 ± 4.39	5.32	1.85	82.39 ± 2.63	3.19	1.72	82.74 ± 4.20	5.08	2.15

Kidney	5.05	4.65 ± 0.33	7.10	−7.92	5.15 ± 0.50	9.71	1.98	4.95 ± 0.29	5.86	−1.98
	10.10	9.15 ± 0.43	4.70	−9.41	11.32 ± 0.67	5.92	12.08	10.23 ± 1.03	10.07	1.29
	25.25	23.42 ± 1.98	8.45	−7.25	26.72 ± 1.28	4.79	5.82	25.76 ± 2.08	8.07	2.02

Brain	5.05	5.01 ± 0.29	5.79	−0.79	4.63 ± 0.52	11.23	−8.32	4.88 ± 0.51	10.45	−3.37
	10.10	10.90 ± 1.49	13.67	7.92	9.90 ± 1.40	14.14	−1.98	11.15 ± 0.78	7.00	10.40
	25.25	26.37 ± 3.36	12.74	4.44	23.03 ± 1.87	8.12	−8.79	24.71 ± 1.25	5.06	−2.14

Stomach	8.10	7.63 ± 0.44	5.77	−5.80	8.25 ± 0.99	12.00	1.85	7.70 ± 0.45	5.84	−4.94
	40.50	43.37 ± 4.04	9.32	7.09	37.46 ± 0.73	1.95	−7.51	42.13 ± 5.48	13.01	4.02
	81.00	79.72 ± 0.23	0.29	−1.58	83.45 ± 7.55	9.05	3.02	80.64 ± 1.53	1.90	−0.44

Uterus	5.05	4.39 ± 0.13	2.96	−13.07	5.12 ± 0.36	7.03	1.39	4.85 ± 0.40	8.25	−3.96
	10.10	9.06 ± 0.64	7.06	−10.30	8.92 ± 0.18	2.02	−11.68	9.19 ± 0.51	5.55	−9.01
	25.25	22.05 ± 1.03	4.67	−12.67	26.33 ± 2.74	10.41	4.28	22.84 ± 0.66	2.89	−9.54

Ovary	5.05	4.70 ± 0.61	12.98	−6.93	4.45 ± 0.66	14.83	−11.88	4.73 ± 0.63	13.32	−6.34
	10.10	10.18 ± 1.02	10.02	0.79	9.05 ± 0.65	7.18	−10.40	9.31 ± 0.61	6.55	−7.82
	25.25	24.37 ± 1.14	4.68	−3.49	24.60 ± 1.54	6.26	−2.57	23.99 ± 1.27	5.29	−4.99

Intestine	8.10	7.82 ± 1.13	14.45	−3.46	8.57 ± 0.95	11.09	5.80	8.13 ± 1.13	13.90	0.37
	40.50	42.25 ± 5.88	13.92	4.32	35.91 ± 1.88	5.24	−11.33	40.48 ± 5.17	12.77	−0.05
	81.00	78.00 ± 9.32	11.95	−3.70	86.10 ± 10.19	11.84	6.30	83.37 ± 11.64	13.96	2.93

**Table 19:** Stability of astragalin in rat tissues (*n* = 6).

Tissues	Spiked (ng/mL)	Room temperature			Freeze-thaw (−20°C, 3 times)			Long term (−20°C, 30 days)		
		Mean ± SD	RSD (%)	RE (%)	Mean ± SD	RSD (%)	RE (%)	Mean ± SD	RSD (%)	RE (%)
Heart	8.10	7.70 ± 0.45	5.84	−4.94	8.47 ± 1.09	12.87	4.57	8.00 ± 0.93	11.63	−1.23
	40.50	36.34 ± 1.61	4.43	−10.27	40.78 ± 3.39	8.31	0.69	39.21 ± 4.04	10.30	−3.19
	81.00	86.71 ± 12.77	14.73	7.05	82.81 ± 4.93	5.95	2.23	88.04 ± 10.68	12.13	8.69

Liver	8.10	8.40 ± 0.49	5.83	3.70	9.25 ± 0.48	5.19	14.20	9.28 ± 1.20	12.93	14.57
	40.50	38.23 ± 1.64	4.29	−5.60	40.11 ± 5.05	12.59	−0.96	37.31 ± 3.82	10.24	−7.88
	81.00	78.97 ± 1.50	1.90	−2.51	86.85 ± 4.39	5.05	7.22	79.81 ± 2.36	2.96	−1.47

Spleen	5.05	4.83 ± 0.62	12.84	−4.36	4.67 ± 0.52	11.13	−7.52	5.08 ± 0.25	4.92	0.59
	10.10	10.65 ± 0.66	6.20	5.45	10.39 ± 1.08	10.39	2.87	11.05 ± 1.42	12.85	9.41
	25.25	23.18 ± 3.01	12.99	−8.20	26.33 ± 2.81	10.67	4.28	25.37 ± 3.04	11.98	0.48

Lung	8.10	9.00 ± 1.11	12.33	11.11	8.58 ± 0.72	8.39	5.93	8.59 ± 0.91	10.59	6.05
	40.50	41.43 ± 3.85	9.29	2.30	44.81 ± 4.50	10.04	10.64	43.01 ± 3.91	9.09	6.20
	81.00	86.86 ± 7.48	8.61	7.23	83.80 ± 6.72	8.02	3.46	90.25 ± 1.68	1.86	11.42

Kidney	5.05	4.43 ± 0.47	10.61	−12.28	4.90 ± 0.60	12.24	−2.97	4.53 ± 0.53	11.70	−10.30
	10.10	9.49 ± 0.84	8.85	−6.04	10.99 ± 1.25	11.37	8.81	10.40 ± 1.02	9.81	2.97
	25.25	22.06 ± 1.98	8.98	−12.63	26.65 ± 1.89	7.09	5.54	23.32 ± 3.02	12.95	−7.64

Brain	5.05	4.48 ± 0.59	13.17	−11.29	5.44 ± 0.52	9.56	7.72	4.82 ± 0.42	8.71	−4.55
	10.10	9.00 ± 0.76	8.44	−10.89	9.87 ± 1.46	14.79	−2.28	9.59 ± 0.74	7.72	−5.05
	25.25	23.04 ± 2.82	12.24	−8.75	23.76 ± 3.54	14.90	−5.90	21.71 ± 0.54	2.49	−14.02

Stomach	8.10	8.49 ± 0.69	8.13	4.81	9.30 ± 1.33	14.30	14.81	9.01 ± 0.59	6.55	11.23
	81.00	93.14 ± 4.03	4.33	14.99	79.09 ± 1.25	1.58	−2.36	87.46 ± 7.78	8.90	7.98
	162.00	156.45 ± 23.16	14.80	−3.43	152.89 ± 21.65	14.16	−5.62	154.82 ± 22.74	14.69	−4.43

Uterus	5.05	4.37 ± 0.34	7.78	−13.47	5.23 ± 0.49	9.37	3.56	4.52 ± 0.52	11.50	−10.50
	10.10	9.18 ± 0.78	8.50	−9.11	11.12 ± 1.40	12.59	10.10	10.23 ± 1.09	10.65	1.29
	25.25	23.30 ± 2.05	8.80	−7.72	28.41 ± 1.48	5.21	12.51	26.18 ± 3.07	11.73	3.68

Ovary	8.10	9.05 ± 1.07	11.82	11.73	9.15 ± 1.34	14.64	12.96	8.60 ± 1.19	13.84	6.17
	40.50	37.87 ± 4.17	11.01	−6.49	45.76 ± 5.66	12.37	12.99	42.26 ± 6.03	14.27	4.35
	81.00	82.74 ± 12.23	14.78	2.15	92.04 ± 8.01	8.70	13.63	92.04 ± 11.52	12.52	13.63

Intestine	8.10	8.28 ± 1.11	13.41	2.22	8.99 ± 0.78	8.68	10.99	8.85 ± 0.81	9.15	9.26
	40.50	40.39 ± 5.04	12.48	−0.27	36.13 ± 1.00	2.77	−10.79	39.29 ± 5.25	13.36	−2.99
	81.00	83.65 ± 9.85	11.78	3.27	81.48 ± 9.48	11.63	0.59	85.26 ± 7.09	8.32	5.26

**Table 20:** Stability of naringin in rat tissues (*n* = 6).

Tissues	Spiked (ng/mL)	Room temperature			Freeze-thaw (−20°C, 3 times)			Long term (−20°C, 30 days)		
		Mean ± SD	RSD (%)	RE (%)	Mean ± SD	RSD (%)	RE (%)	Mean ± SD	RSD (%)	RE (%)
Heart	8.10	8.18 ± 0.99	12.10	0.99	9.31 ± 0.59	6.34	14.94	8.34 ± 1.05	12.59	2.96
	405.00	398.76 ± 53.76	13.48	−1.54	363.13 ± 28.56	7.86	−10.34	392.60 ± 57.37	14.61	−3.06
	810.00	829.59 ± 77.67	9.36	2.42	842.66 ± 90.17	10.70	4.03	833.28 ± 79.23	9.51	2.87

Liver	12.20	12.40 ± 1.51	12.18	1.64	11.43 ± 0.64	5.60	−6.31	11.77 ± 1.76	14.95	−3.52
	1,220.00	1,109.92 ± 162.72	14.66	−9.02	1,056.84 ± 59.00	5.58	−13.37	1,148.25 ± 169.62	14.77	−5.88
	4,880.00	4,705.83 ± 702.90	14.94	−3.57	5,273.67 ± 504.56	9.57	8.07	4,969.00 ± 514.84	10.36	1.82

Spleen	8.10	8.85 ± 1.08	12.20	9.26	9.05 ± 1.22	13.48	11.73	8.07 ± 0.85	10.53	−0.37
	810.00	801.57 ± 45.20	5.64	−1.04	848.96 ± 85.32	10.05	4.81	822.63 ± 46.56	5.66	1.56
	4,050.00	4,143.06 ± 577.07	13.93	2.30	3,865.82 ± 370.26	9.58	−4.55	4,201.39 ± 602.33	14.34	3.74

Lung	8.10	9.26 ± 1.01	10.91	14.32	8.77 ± 0.40	4.56	8.27	9.03 ± 1.06	11.74	11.48
	810.00	841.43 ± 64.45	7.66	3.88	849.40 ± 106.55	12.54	4.86	867.33 ± 105.64	12.18	7.08
	4,050.00	3,705.01 ± 526.75	14.22	−8.52	4,317.76 ± 550.38	12.75	6.61	3,697.64 ± 514.32	13.91	−8.70

Kidney	8.10	9.15 ± 1.10	12.02	12.96	8.31 ± 0.48	5.78	2.59	9.31 ± 1.02	10.96	14.94
	810.00	892.39 ± 40.99	4.59	10.17	834.96 ± 117.60	14.08	3.08	846.20 ± 106.88	12.63	4.47
	4,050.00	4,133.40 ± 608.45	14.72	2.06	3,779.16 ± 448.50	11.87	−6.69	3,747.32 ± 516.20	13.78	−7.47

Brain	8.10	8.35 ± 1.01	12.10	3.09	7.92 ± 1.04	13.13	2.22	7.49 ± 0.50	6.68	−7.53
	405.00	444.59 ± 47.61	10.71	9.78	385.42 ± 41.47	10.76	−4.83	424.79 ± 47.09	11.09	4.89
	810.00	882.19 ± 104.39	11.83	8.91	817.76 ± 120.70	14.76	0.96	810.79 ± 121.05	14.93	0.10

Stomach	8.10	8.47 ± 1.21	14.29	4.57	9.19 ± 1.28	13.93	13.46	7.86 ± 0.52	6.62	−2.96
	810.00	790.05 ± 106.47	13.48	−2.46	777.09 ± 71.41	9.19	−4.06	842.24 ± 101.48	12.05	3.98
	8,100.00	8,149.70 ± 564.58	6.93	0.61	7,454.36 ± 368.62	4.95	−7.97	7,672.71 ± 291.70	3.80	−5.28

Uterus	8.10	8.96 ± 0.64	7.14	10.62	9.09 ± 1.25	13.75	12.22	9.14 ± 0.94	10.28	12.84
	1,215.00	1,155.91 ± 151.48	13.10	−4.86	1,358.76 ± 84.48	6.22	11.83	1,266.07 ± 11.09	0.88	4.20
	2,430.00	2,461.22 ± 348.52	14.16	1.28	2,262.54 ± 87.25	3.86	−6.89	2,330.98 ± 332.29	14.26	−4.07

Ovary	8.10	8.06 ± 1.07	13.28	−0.49	9.16 ± 0.74	8.08	13.09	7.97 ± 1.05	13.17	−1.60
	810.00	820.47 ± 26.50	3.23	1.29	895.06 ± 54.93	6.14	10.50	827.50 ± 32.71	3.95	2.16
	4,050.00	3,954.66 ± 490.84	12.41	−2.35	4,095.99 ± 558.04	13.62	1.14	4,080.56 ± 377.08	9.24	0.75

Intestine	8.10	8.19 ± 0.90	10.99	1.11	9.30 ± 1.24	13.33	14.81	7.63 ± 0.13	1.70	−5.80
	810.00	818.79 ± 115.79	14.14	1.09	877.24 ± 105.43	12.02	8.30	845.43 ± 80.79	9.56	4.37
	8,100.00	8,351.44 ± 1,228.10	14.71	3.10	8,229.70 ± 1,151.14	13.99	1.60	8,818.55 ± 1,318.14	14.95	8.87

**Table 21:** Stability of eriodictyol in rat tissues (*n* = 6).

Tissues	Spiked (ng/mL)	Room temperature			Freeze-thaw (−20°C, 3 times)			Long term (−20°C, 30 days)		
		Mean ± SD	RSD (%)	RE (%)	Mean ± SD	RSD (%)	RE (%)	Mean ± SD	RSD (%)	RE (%)
Liver	8.10	8.74 ± 0.86	9.84	7.90	9.07 ± 1.31	14.44	11.98	7.90 ± 0.98	12.41	−2.47
	40.50	35.14 ± 2.55	7.26	−13.23	41.26 ± 2.48	6.01	1.88	37.37 ± 2.39	6.40	−7.73
	81.00	86.70 ± 8.92	10.29	7.04	81.90 ± 12.13	14.81	1.11	91.19 ± 9.55	10.47	12.58

Spleen	5.05	4.58 ± 0.65	14.19	−9.31	5.23 ± 0.16	3.06	3.56	4.82 ± 0.61	12.66	−4.55
	10.10	11.14 ± 1.33	11.94	10.30	10.87 ± 0.46	4.23	7.62	11.35 ± 1.08	9.52	12.38
	25.25	24.57 ± 2.36	9.61	−2.69	21.75 ± 1.80	8.28	−13.86	24.87 ± 1.86	7.48	−1.50

Lung	8.10	8.67 ± 0.94	10.84	7.04	8.52 ± 1.06	12.44	5.19	8.94 ± 0.99	11.07	10.37
	40.50	44.21 ± 6.02	13.62	9.16	38.47 ± 3.91	10.16	−5.01	40.58 ± 5.08	12.52	0.20
	81.00	74.39 ± 7.67	10.31	−8.16	88.31 ± 8.37	9.48	9.02	81.83 ± 5.22	6.38	1.02

Stomach	8.10	8.07 ± 0.83	10.29	−0.37	9.21 ± 0.21	2.28	13.70	8.95 ± 0.94	10.50	10.49
	81.00	76.31 ± 4.44	5.82	−5.79	87.52 ± 3.29	3.76	8.05	80.47 ± 3.35	4.16	−0.65
	162.00	156.23 ± 14.25	9.12	−3.56	163.44 ± 23.11	14.14	0.89	165.97 ± 24.01	14.47	2.45

Uterus	5.05	5.34 ± 0.79	14.79	5.74	5.15 ± 0.41	7.96	1.98	4.96 ± 0.67	13.51	−1.78
	10.10	10.77 ± 1.36	12.63	6.63	9.56 ± 1.35	14.12	−5.35	10.42 ± 0.87	8.35	3.17
	25.25	24.07 ± 3.23	13.42	−4.67	22.30 ± 2.21	9.91	−11.68	22.60 ± 3.35	14.82	−10.50

Ovary	8.10	8.99 ± 1.10	12.24	10.99	8.83 ± 1.25	14.16	9.01	9.25 ± 0.73	7.89	14.20
	40.50	37.31 ± 1.78	4.77	−7.88	39.47 ± 5.77	14.62	−2.54	40.46 ± 6.01	14.85	−0.10
	81.00	89.65 ± 10.06	11.22	10.68	84.97 ± 6.08	7.16	4.90	86.94 ± 8.45	9.72	7.33

Intestine	8.10	8.73 ± 1.15	13.17	7.78	8.95 ± 1.15	12.85	10.49	9.31 ± 1.23	13.21	14.94
	81.00	82.71 ± 11.00	13.30	2.11	77.65 ± 10.42	13.42	−4.14	77.44 ± 10.21	13.18	−4.40
	162.00	159.30 ± 2.85	1.79	−1.67	181.74 ± 15.06	8.29	12.19	164.51 ± 10.80	6.56	1.55

**Table 22:** Stability of naringenin in rat tissues (*n* = 6).

Tissues	Spiked (ng/mL)	Room temperature			Freeze-thaw (−20°C, 3 times)			Long term (−20°C, 30 days)		
		Mean ± SD	RSD (%)	RE (%)	Mean ± SD	RSD (%)	RE (%)	Mean ± SD	RSD (%)	RE (%)
Heart	8.10	8.04 ± 1.04	12.94	−0.74	8.24 ± 1.17	14.20	1.73	8.00 ± 1.08	13.50	−1.23
	81.00	81.52 ± 11.82	14.50	0.64	84.33 ± 6.52	7.73	4.11	77.37 ± 6.28	8.12	−4.48
	162.00	151.00 ± 20.37	13.49	−6.79	169.65 ± 21.47	12.66	4.72	171.11 ± 25.44	14.87	5.62

Liver	8.10	8.00 ± 0.80	10.00	−1.23	9.27 ± 1.32	14.24	14.44	8.55 ± 1.25	14.62	5.56
	405.00	418.93 ± 47.87	11.43	3.44	436.27 ± 64.51	14.79	7.72	434.87 ± 44.93	10.33	7.38
	810.00	778.74 ± 112.79	14.48	−3.86	771.71 ± 87.80	11.38	−4.73	734.04 ± 37.39	5.09	−9.38

Spleen	8.10	8.58 ± 0.70	8.16	5.93	9.22 ± 0.83	9.00	13.83	8.87 ± 1.19	13.42	9.51
	81.00	80.48 ± 6.41	7.96	−0.64	90.38 ± 0.78	0.86	11.58	83.50 ± 8.26	9.89	3.09
	162.00	139.60 ± 17.83	12.77	−13.83	165.50 ± 7.38	4.46	2.16	156.36 ± 14.57	9.32	−3.48

Lung	8.10	9.15 ± 1.10	12.02	12.96	7.98 ± 0.83	10.40	−1.48	9.31 ± 1.22	13.10	14.98
	81.00	74.57 ± 7.11	9.53	−7.94	84.78 ± 8.89	10.49	4.67	79.09 ± 8.84	11.18	−2.36
	162.00	156.47 ± 22.72	14.52	−3.41	154.67 ± 21.84	14.12	−4.52	167.06 ± 23.04	13.79	3.12

Kidney	8.10	8.45 ± 1.14	13.49	4.32	8.42 ± 1.06	12.59	3.95	8.13 ± 1.09	13.41	0.37
	81.00	77.29 ± 9.32	12.06	−4.58	86.85 ± 6.12	7.05	7.22	83.16 ± 11.44	13.76	2.67
	162.00	167.81 ± 24.47	14.58	3.59	159.11 ± 23.36	14.68	−1.78	162.72 ± 22.74	13.97	0.44

Brain	5.05	4.56 ± 0.49	10.75	−9.70	4.88 ± 0.64	13.11	−3.37	4.71 ± 0.42	8.92	−6.73
	10.10	11.45 ± 1.03	9.00	13.37	10.80 ± 0.98	9.07	6.93	10.81 ± 1.45	13.41	7.03
	25.25	23.97 ± 2.89	12.06	−5.07	28.86 ± 1.83	6.34	14.30	25.78 ± 1.99	7.72	2.10

Stomach	8.10	8.16 ± 0.84	10.29	0.74	8.84 ± 0.84	9.50	9.14	8.35 ± 1.16	13.89	3.09
	405.00	407.95 ± 57.80	14.17	0.73	417.68 ± 62.09	14.87	3.13	435.78 ± 65.10	14.94	7.60
	810.00	843.25 ± 105.05	12.46	4.10	823.51 ± 95.20	11.56	1.67	881.58 ± 126.68	14.37	8.84

Uterus	8.10	9.06 ± 0.83	9.16	11.85	7.79 ± 0.82	10.53	−3.83	8.81 ± 1.28	14.53	8.77
	81.00	73.81 ± 5.18	7.02	−8.88	85.29 ± 11.93	13.99	5.30	73.36 ± 5.25	7.16	−9.43
	162.00	153.95 ± 20.86	13.55	−4.97	172.19 ± 17.57	10.20	6.29	154.24 ± 22.37	14.50	−4.79

Ovary	8.10	7.86 ± 0.67	8.52	−2.96	8.73 ± 1.17	13.40	7.78	7.76 ± 0.50	6.44	−4.20
	81.00	78.53 ± 11.16	14.21	−3.05	86.02 ± 9.25	10.75	6.20	76.30 ± 7.87	10.31	−5.80
	162.00	144.08 ± 10.08	7.00	−11.06	183.49 ± 8.80	4.80	13.27	156.39 ± 23.1	14.77	−3.46

Intestine	8.10	7.69 ± 1.01	13.13	−5.06	7.59 ± 0.54	7.11	−6.30	8.16 ± 0.93	11.40	0.74
	405.00	409.72 ± 54.55	13.31	1.17	399.15 ± 58.51	14.66	−1.44	434.72 ± 60.96	14.02	7.34
	810.00	823.39 ± 83.78	10.18	1.65	717.09 ± 36.44	5.08	−11.47	748.90 ± 78.81	10.52	−7.54

**Table 23:** Stability of kaempferol in rat tissues (*n* = 6).

Tissues	Spiked (ng/mL)	Room temperature			Freeze-thaw (−20°C, 3 times)			Long term (−20°C, 30 days)		
		Mean ± SD	RSD (%)	RE (%)	Mean ± SD	RSD (%)	RE (%)	Mean ± SD	RSD (%)	RE (%)
Liver	8.10	8.39 ± 1.60	14.04	3.54	8.62 ± 1.09	12.69	6.46	8.57 ± 1.42	14.62	5.76
	40.50	43.77 ± 9.22	13.06	8.08	50.17 ± 2.46	4.90	13.88	46.07 ± 9.62	14.87	13.75
	81.00	83.39 ± 13.38	14.35	2.95	82.41 ± 11.32	13.74	1.74	88.11 ± 13.51	14.33	8.78

Spleen	12.20	11.80 ± 1.91	14.15	−3.28	10.33 ± 0.84	8.09	−14.36	12.19 ± 1.43	11.75	−0.11
	91.50	89.37 ± 5.89	6.59	−2.32	104.78 ± 13.69	13.06	14.52	87.15 ± 2.32	2.66	−4.76
	183.00	189.62 ± 32.89	14.35	3.62	188.06 ± 24.01	12.77	2.77	201.14 ± 33.04	14.43	9.91

Lung	5.05	5.26 ± 0.82	14.68	4.16	4.91 ± 0.45	9.19	−2.84	5.11 ± 0.97	14.94	1.19
	10.10	8.89 ± 0.72	8.05	−11.98	9.79 ± 1.73	14.71	−3.07	9.07 ± 0.74	8.20	−10.23
	25.25	23.20 ± 5.65	14.34	−8.11	24.29 ± 3.55	14.60	−3.79	24.46 ± 6.43	14.31	−3.13

Kidney	12.20	10.56 ± 1.22	11.55	−13.42	10.86 ± 1.26	11.55	−10.96	10.39 ± 1.07	10.34	−14.86
	42.70	41.76 ± 4.97	11.90	−2.20	40.96 ± 5.41	13.21	−4.07	43.77 ± 5.68	12.99	2.51
	85.40	86.58 ± 7.17	8.28	1.39	86.17 ± 11.23	13.04	0.90	91.18 ± 8.09	8.88	6.76

Stomach	5.05	4.77 ± 1.03	11.50	−5.48	4.86 ± 0.38	7.85	−3.83	5.05 ± 0.64	12.74	−0.07
	10.10	11.62 ± 1.09	9.42	14.02	9.41 ± 0.37	3.95	−6.80	10.40 ± 1.12	10.79	2.94
	25.25	24.02 ± 2.30	9.55	−4.87	24.27 ± 4.81	14.82	−3.89	25.50 ± 4.23	14.58	1.00

Uterus	5.05	4.42 ± 0.24	5.35	−12.48	5.37 ± 0.09	1.69	6.27	4.74 ± 0.51	10.80	−6.20
	10.10	11.14 ± 1.60	14.33	10.26	9.57 ± 0.75	7.87	−5.25	10.75 ± 1.57	14.60	6.40
	25.25	22.74 ± 3.64	14.02	−9.95	25.59 ± 0.85	3.31	1.36	25.10 ± 2.16	8.61	−0.58

Intestine	8.10	8.36 ± 1.13	13.49	3.17	8.27 ± 1.20	14.53	2.06	8.54 ± 1.33	14.62	5.43
	121.50	97.69 ± 2.61	2.67	−14.60	112.52 ± 13.53	12.03	−7.39	100.47 ± 2.96	2.95	−14.31
	243.00	241.99 ± 28.92	11.95	−0.42	286.61 ± 39.49	13.78	14.95	265.03 ± 42.79	14.15	9.07

**Table 24:** Tissue concentrations of seven analytes at different times after oral administration in 8-month-old rats (ng/g, *n* = 5).

Analytes	Tissue	0.25 h	0.5 h	1 h	2 h	4 h	8 h	12 h
Neoeriocitrin	Heart	39.18 ± 10.58	222.17 ± 35.38	173.93 ± 32.14	121.81 ± 30.37	68.13 ± 16.59	9.22 ± 1.68	0.00
	Liver	31.86 ± 7.17	501.02 ± 59.13	3,247.80 ± 413.47	2,201.25 ± 211.27	355.59 ± 119.14	167.65 ± 49.30	14.25 ± 6.75
	Spleen	102.90 ± 13.98	2,190.70 ± 345.97	8,008.91 ± 715.34	1,397.10 ± 286.06	159.13 ± 56.30	36.68 ± 34.51	7.50 ± 1.74
	Lung	1,119.98 ± 146.91	6,039.56 ± 279.40	7,837.99 ± 493.16	5,080.60 ± 431.81	695.30 ± 107.71	34.26 ± 8.21	0.00
	Kidney	134.72 ± 9.14	414.94 ± 60.69	257.50 ± 75.86	112.65 ± 30.13	29.32 ± 9.44	7.65 ± 4.81	0.00
	Brain	13.32 ± 2.84	25.03 ± 2.27	34.04 ± 6.39	8.31 ± 0.92	4.45 ± 1.87	0.00	0.00
	Stomach	4,061.88 ± 312.91	7,324.59 ± 397.44	3,775.38 ± 465.61	1,513.07 ± 264.13	502.61 ± 189.18	25.85 ± 5.70	0.00
	Uterus	75.22 ± 10.79	156.79 ± 16.29	251.62 ± 35.33	70.32 ± 13.94	26.97 ± 4.04	7.26 ± 2.46	0.00
	Ovary	103.83 ± 17.25	162.03 ± 24.58	251.99 ± 32.24	146.23 ± 16.34	12.27 ± 6.13	0.00	0.00
	Intestine	188.58 ± 45.77	4,818.76 ± 299.03	8,133.84 ± 581.81	993.61 ± 114.13	781.08 ± 121.90	163.04 ± 32.57	0.00

Luteolin-7-*O-β-*D-glucoside	Heart	9.58 ± 2.01	20.71 ± 3.14	15.06 ± 2.10	8.87 ± 2.14	3.27 ± 1.06	0.00	0.00
	Liver	7.44 ± 1.19	21.80 ± 4.17	12.38 ± 2.34	6.65 ± 1.14	3.48 ± 2.53	0.00	0.00
	Spleen	4.11 ± 2.23	7.69 ± 3.58	38.03 ± 7.51	15.62 ± 6.06	7.51 ± 2.77	2.48 ± 1.85	0.00
	Lung	8.82 ± 1.26	24.53 ± 2.02	51.71 ± 3.02	20.08 ± 4.13	7.69 ± 2.51	2.82 ± 3.26	0.00
	Kidney	2.43 ± 1.72	4.81 ± 2.61	8.44 ± 6.55	32.29 ± 9.01	17.40 ± 5.39	2.88 ± 1.44	0.00
	Brain	2.14 ± 0.87	13.17 ± 1.79	7.14 ± 0.92	1.95 ± 0.74	1.05 ± 0.52	0.00	0.00
	Stomach	70.44 ± 7.01	92.66 ± 11.08	64.82 ± 5.00	46.88 ± 9.75	31.71 ± 6.82	3.79 ± 2.90	0.00
	Uterus	8.90 ± 0.95	9.65 ± 0.59	22.33 ± 4.67	6.52 ± 2.08	3.29 ± 2.27	0.00	0.00
	Ovary	6.09 ± 1.20	9.94 ± 3.64	17.25 ± 6.60	20.16 ± 5.62	4.65 ± 2.56	0.00	0.00
	Intestine	21.13 ± 3.16	87.42 ± 11.09	68.78 ± 8.31	40.51 ± 7.63	21.03 ± 7.04	2.71 ± 1.35	0.00

Astragalin	Heart	5.16 ± 2.00	44.98 ± 13.39	21.10 ± 3.34	9.17 ± 1.97	4.55 ± 1.51	1.34 ± 0.67	0.00
	Liver	9.55 ± 1.67	24.38 ± 5.93	63.85 ± 10.04	22.40 ± 3.22	8.48 ± 3.52	4.53 ± 3.04	0.00
	Spleen	4.82 ± 0.94	11.88 ± 2.34	23.04 ± 3.18	41.29 ± 6.27	8.69 ± 4.48	3.32 ± 2.61	0.00
	Lung	9.26 ± 1.67	12.71 ± 1.47	44.28 ± 6.91	31.32 ± 4.41	15.46 ± 5.84	8.06 ± 2.45	2.04 ± 1.02
	Kidney	6.04 ± 1.45	8.64 ± 2.33	13.76 ± 1.92	19.11 ± 1.64	7.87 ± 2.20	2.70 ± 2.12	0.00
	Brain	5.41 ± 1.71	6.08 ± 1.44	27.72 ± 7.78	14.98 ± 1.23	0.13 ± 0.07	0.00	0.00
	Stomach	54.36 ± 13.29	92.09 ± 5.08	124.20 ± 20.11	242.33 ± 28.28	90.80 ± 5.12	12.87 ± 3.39	0.00
	Uterus	9.17 ± 1.60	12.51 ± 1.46	36.06 ± 14.35	21.44 ± 8.70	8.11 ± 1.98	4.13 ± 2.88	0.00
	Ovary	16.09 ± 2.13	41.80 ± 7.89	56.24 ± 5.64	35.79 ± 7.01	6.55 ± 2.67	1.27 ± 0.64	0.00
	Intestine	9.08 ± 2.60	13.45 ± 5.10	46.33 ± 14.85	84.05 ± 6.62	54.11 ± 16.00	0.00	0.00

Naringin	Heart	24.05 ± 3.55	305.07 ± 23.30	802.23 ± 48.96	95.19 ± 19.18	25.10 ± 8.37	18.89 ± 6.74	4.22 ± 3.00
	Liver	3,100.99 ± 329.24	6,844.61 ± 394.09	8,990.54 ± 645.09	4,696.73 ± 787.00	2,309.32 ± 361.78	4,316.78 ± 750.95	87.26 ± 61.30
	Spleen	1,033.31 ± 123.04	2,117.77 ± 261.76	8,347.29 ± 550.68	7,318.88 ± 584.70	4,566.69 ± 415.28	161.26 ± 73.58	65.26 ± 20.78
	Lung	2,403.72 ± 542.57	6,136.89 ± 597.95	8,028.23 ± 577.45	4,758.34 ± 388.04	2,218.44 ± 480.61	261.11 ± 187.44	72.21 ± 44.57
	Kidney	3,898.03 ± 305.12	5,952.40 ± 564.86	6,815.11 ± 427.58	3,831.32 ± 409.31	997.11 ± 113.99	182.31 ± 72.30	10.28 ± 2.11
	Brain	254.15 ± 31.88	914.44 ± 114.98	769.77 ± 106.65	632.54 ± 64.55	403.25 ± 46.45	163.38 ± 36.62	13.74 ± 11.25
	Stomach	1,089.48 ± 158.52	2,523.96 ± 367.74	4,874.73 ± 712.92	6,845.44 ± 660.93	1,957.66 ± 275.96	205.47 ± 86.57	6.92 ± 3.84
	Uterus	1,797.02 ± 178.68	3,515.04 ± 532.80	4,022.84 ± 195.00	3,052.92 ± 310.56	1,130.50 ± 192.81	2,476.23 ± 917.77	38.34 ± 29.09
	Ovary	3,884.52 ± 446.17	5,049.08 ± 241.23	8,319.83 ± 564.53	2,283.59 ± 417.38	1,434.68 ± 357.23	113.37 ± 34.10	0.00
	Intestine	1,192.96 ± 191.76	4,933.79 ± 803.89	8,510.36 ± 952.27	11,753.34 ± 1,203.70	1,465.91 ± 469.36	448.75 ± 196.23	0.00

Eriodictyol	Liver	19.37 ± 2.97	23.76 ± 2.79	144.70 ± 20.72	71.92 ± 16.12	136.87 ± 30.56	96.07 ± 48.74	0.00
	Spleen	0.00	4.29 ± 1.55	19.26 ± 1.84	26.20 ± 4.64	2.11 ± 0.23	0.00	0.00
	Lung	49.46 ± 6.08	30.50 ± 7.01	19.56 ± 2.94	8.70 ± 1.76	3.87 ± 2.91	0.00	0.00
	Stomach	58.57 ± 7.99	89.49 ± 17.62	105.23 ± 20.57	148.36 ± 15.79	80.59 ± 7.28	13.04 ± 4.34	0.00
	Uterus	8.46 ± 1.82	16.52 ± 4.19	33.10 ± 4.66	8.25 ± 5.11	1.08 ± 0.06	0.00	0.00
	Ovary	6.21 ± 0.72	10.50 ± 1.41	32.09 ± 2.87	10.05 ± 1.43	4.08 ± 1.95	0.00	0.00
	Intestine	37.69 ± 12.93	138.00 ± 61.75	209.76 ± 71.61	69.79 ± 20.12	26.10 ± 9.96	6.25 ± 1.06	0.00

Naringenin	Heart	2.45 ± 0.78	5.15 ± 1.12	7.68 ± 1.07	21.54 ± 3.80	29.00 ± 5.82	182.90 ± 11.46	0.00
	Liver	17.88 ± 2.93	31.73 ± 6.68	46.63 ± 5.29	155.17 ± 25.16	761.78 ± 99.66	837.21 ± 69.13	23.80 ± 12.62
	Spleen	11.50 ± 1.36	16.51 ± 3.35	30.39 ± 11.29	165.29 ± 11.28	97.20 ± 18.16	57.48 ± 13.95	11.16 ± 9.47
	Lung	11.66 ± 1.74	37.15 ± 6.87	63.24 ± 4.93	53.05 ± 7.00	48.79 ± 12.05	101.33 ± 17.69	3.31 ± 0.63
	Kidney	8.65 ± 2.02	39.65 ± 4.97	54.99 ± 7.07	48.80 ± 7.84	53.29 ± 8.58	103.53 ± 14.66	0.00
	Brain	3.27 ± 1.49	9.39 ± 3.66	5.01 ± 1.91	0.80 ± 0.61	0.00	0.00	0.00
	Stomach	234.16 ± 20.86	313.03 ± 55.49	369.35 ± 40.89	622.55 ± 25.86	435.45 ± 91.57	162.98 ± 18.45	8.57 ± 1.15
	Uterus	17.51 ± 3.47	24.28 ± 4.26	141.20 ± 18.48	79.43 ± 14.57	182.01 ± 20.39	118.06 ± 33.90	8.92 ± 1.85
	Ovary	16.78 ± 5.55	28.05 ± 7.04	132.19 ± 34.53	75.75 ± 9.82	185.35 ± 10.12	137.40 ± 23.25	0.00
	Intestine	13.35 ± 2.46	31.49 ± 14.73	140.07 ± 31.41	60.03 ± 15.27	144.57 ± 40.01	98.08 ± 45.70	0.00

Kaempferol	Liver	0.67 ± 0.35	11.53 ± 4.46	68.82 ± 13.01	49.80 ± 14.43	17.44 ± 5.63	2.51 ± 1.60	0.00
	Spleen	0.00	9.67 ± 3.02	182.98 ± 17.07	22.46 ± 3.82	16.06 ± 6.56	8.96 ± 7.29	0.00
	Lung	11.40 ± 1.07	24.08 ± 2.27	29.52 ± 1.85	19.73 ± 1.14	8.78 ± 2.16	0.87 ± 0.74	0.00
	Kidney	16.19 ± 3.45	24.34 ± 5.27	31.90 ± 5.32	55.28 ± 5.99	22.71 ± 4.80	0.00	0.00
	Stomach	17.25 ± 1.93	24.54 ± 2.69	39.83 ± 13.60	43.81 ± 5.36	21.79 ± 7.01	4.95 ± 3.58	0.00
	Uterus	3.36 ± 1.70	6.19 ± 0.85	24.01 ± 3.45	18.35 ± 3.34	12.50 ± 1.60	0.00	0.00
	Intestine	44.75 ± 8.17	80.12 ± 14.32	405.27 ± 37.64	348.26 ± 12.33	310.25 ± 42.39	39.37 ± 8.12	0.00

**Table 25:** Tissue concentrations of seven analytes at different times after oral administration in 18-month-old rats (ng/g, *n* = 5).

Analytes	Tissue	0.25 h	0.5 h	1 h	2 h	4 h	8 h	12 h
Neoeriocitrin	Heart	31.73 ± 3.60	189.07 ± 21.88	164.81 ± 17.36	101.18 ± 12.35	77.21 ± 5.02	25.17 ± 2.90	0.00
	Liver	16.96 ± 1.53	401.00 ± 31.39	2,431.18 ± 217.03	1,571.02 ± 164.80	677.16 ± 48.76	305.28 ± 35.76	0.00
	Spleen	230.11 ± 15.95	2,006.98 ± 117.28	5,970.17 ± 737.71	2,543.47 ± 175.93	1,114.32 ± 88.74	377.12 ± 35.87	0.00
	Lung	1,927.71 ± 172.26	5,092.49 ± 130.22	6,223.83 ± 597.97	4,985.02 ± 490.80	1,463.71 ± 179.74	372.35 ± 38.97	0.00
	Kidney	97.28 ± 8.59	387.12 ± 44.81	235.05 ± 27.59	127.05 ± 7.19	66.06 ± 8.01	13.80 ± 1.59	0.00
	Brain	8.15 ± 0.75	20.59 ± 1.42	24.98 ± 1.99	7.41 ± 0.70	6.47 ± 0.47	0.00	0.00
	Stomach	3,632.23 ± 336.19	5,552.27 ± 384.05	3,964.81 ± 315.74	2,299.33 ± 278.83	1,039.50 ± 119.57	585.36 ± 46.62	68.88 ± 6.55
	Uterus	49.22 ± 4.45	106.19 ± 8.31	180.98 ± 16.16	78.08 ± 8.19	40.89 ± 2.94	17.19 ± 2.02	0.00
	Ovary	78.22 ± 4.57	111.62 ± 10.33	170.29 ± 11.78	138.69 ± 11.04	64.70 ± 6.15	41.51 ± 3.00	0.00
	Intestine	1,646.14 ± 96.19	4,254.15 ± 393.76	6,236.26 ± 431.37	3,118.61 ± 248.35	1,426.29 ± 102.70	463.22 ± 54.26	0.00

Luteolin- 7-*O-β-*D-glucoside	Heart	10.88 ± 1.03	49.05 ± 4.38	29.95 ± 0.77	12.27 ± 1.17	6.94 ± 0.68	6.38 ± 0.78	0.00
	Liver	6.98 ± 0.48	21.34 ± 1.25	48.17 ± 4.46	27.24 ± 1.18	16.49 ± 1.31	9.14 ± 2.54	3.79 ± 3.17
	Spleen	4.95 ± 0.36	12.27 ± 0.69	22.73 ± 2.76	38.76 ± 4.46	14.50 ± 1.15	5.44 ± 0.52	0.00
	Lung	8.68 ± 0.64	11.67 ± 0.66	24.51 ± 2.97	34.10 ± 3.92	13.35 ± 1.63	7.91 ± 0.51	0.00
	Kidney	4.68 ± 0.37	6.35 ± 0.57	11.03 ± 1.16	15.63 ± 1.08	9.87 ± 0.79	6.05 ± 0.58	0.00
	Brain	3.80 ± 0.34	9.14 ± 1.06	25.02 ± 2.94	12.37 ± 0.95	5.11 ± 0.57	0.00	0.00
	Stomach	39.57 ± 4.58	75.86 ± 8.90	130.26 ± 10.04	205.52 ± 22.86	136.00 ± 13.96	26.54 ± 3.51	14.21 ± 1.59
	Uterus	7.72 ± 0.74	10.10 ± 0.99	24.21 ± 2.97	19.84 ± 1.58	12.60 ± 1.20	6.98 ± 0.51	0.00
	Ovary	14.85 ± 1.16	41.55 ± 3.71	38.08 ± 3.99	30.21 ± 3.97	11.18 ± 1.15	0.00	0.00
	Intestine	7.72 ± 0.69	8.97 ± 0.23	38.79 ± 3.71	701.13 ± 6.90	48.59 ± 5.89	14.75 ± 1.70	0.00

Astragalin	Heart	6.39 ± 0.67	21.86 ± 2.21	13.27 ± 1.19	10.78 ± 0.84	5.80 ± 0.52	4.42 ± 0.50	0.00
	Liver	6.93 ± 0.61	18.64 ± 2.16	14.38 ± 1.69	9.51 ± 0.73	7.18 ± 0.80	5.31 ± 1.13	0.00
	Spleen	2.77 ± 0.16	4.42 ± 0.41	30.81 ± 2.13	17.57 ± 1.40	9.09 ± 0.86	6.04 ± 0.44	0.00
	Lung	7.85 ± 0.79	20.13 ± 1.81	39.92 ± 3.12	17.98 ± 1.62	10.30 ± 1.16	7.32 ± 0.82	0.00
	Kidney	2.45 ± 0.17	3.57 ± 0.21	10.31 ± 0.95	34.03 ± 2.35	16.68 ± 1.33	8.76 ± 0.83	3.41 ± 0.25
	Brain	3.23 ± 0.37	10.09 ± 1.17	7.11 ± 0.75	3.84 ± 0.47	2.14 ± 0.14	0.00	0.00
	Stomach	50.47 ± 5.31	73.23 ± 9.63	124.20 ± 20.11	242.33 ± 28.28	90.80 ± 5.12	12.87 ± 3.39	0.00
	Uterus	7.05 ± 0.74	8.07 ± 1.06	15.35 ± 1.58	10.07 ± 1.33	6.87 ± 0.62	4.08 ± 0.46	0.00
	Ovary	5.59 ± 0.59	9.39 ± 1.23	14.14 ± 1.45	17.47 ± 1.79	9.13 ± 1.21	4.01 ± 0.45	0.00
	Intestine	17.15 ± 1.00	73.74 ± 6.83	53.74 ± 3.72	35.12 ± 4.04	15.60 ± 1.24	7.20 ± 0.68	0.00

Naringin	Heart	15.87 ± 2.49	201.48 ± 20.58	643.80 ± 91.40	99.05 ± 15.19	40.88 ± 2.88	22.64 ± 2.88	10.91 ± 1.45
	Liver	2,848.51 ± 428.98	4,590.53 ± 744.43	7,219.96 ± 1,059.27	3,393.97 ± 490.96	1,933.20 ± 259.73	4,047.07 ± 309.31	90.95 ± 10.55
	Spleen	827.46 ± 77.63	1,997.58 ± 151.10	7,005.53 ± 555.82	4,646.08 ± 509.30	3,556.25 ± 666.72	1,077.91 ± 166.30	187.62 ± 28.35
	Lung	2,467.96 ± 219.09	4,314.82 ± 373.65	5,985.15 ± 291.65	3,011.26 ± 247.62	2,118.82 ± 171.12	280.11 ± 33.42	83.92 ± 10.13
	Kidney	3,004.22 ± 241.98	5,728.09 ± 367.43	7,354.37 ± 678.80	3,414.98 ± 295.76	1,578.24 ± 75.40	621.79 ± 17.60	130.97 ± 3.45
	Brain	180.55 ± 11.86	844.75 ± 43.48	756.89 ± 133.87	500.91 ± 16.24	371.38 ± 20.03	127.58 ± 7.22	42.62 ± 2.27
	Stomach	934.78 ± 63.59	2,648.34 ± 29.62	11,647.56 ± 856.48	5,195.02 ± 398.77	2,449.35 ± 120.89	266.57 ± 18.89	8.42 ± 0.65
	Uterus	1,367.90 ± 24.22	3,012.12 ± 11.46	3,367.69 ± 178.65	2,330.25 ± 166.02	1,300.67 ± 153.39	2,066.60 ± 132.32	71.21 ± 3.31
	Ovary	3,842.79 ± 127.02	4,546.92 ± 322.21	6,362.25 ± 267.28	17,813.64 ± 785.27	17,192.38 ± 9,967.81	363.64 ± 29.11	31.74 ± 2.55
	Intestine	673.87 ± 58.98	3,654.42 ± 267.86	6,084.22 ± 648.83	9,323.07 ± 754.96	2,177.045 ± 128.14	965.96 ± 24.06	66.80 ± 4.91

Eriodictyol	Liver	16.96 ± 1.61	30.10 ± 4.27	36.17 ± 5.19	13.66 ± 1.89	8.19 ± 0.65	3.16 ± 2.73	0.00
	Spleen	0.00	11.15 ± 1.26	25.17 ± 2.83	39.09 ± 2.48	0.00	0.00	0.00
	Lung	47.00 ± 3.08	20.90 ± 3.85	15.87 ± 2.04	7.50 ± 0.47	5.48 ± 0.81	0.00	0.00
	Stomach	57.23 ± 5.34	64.35 ± 9.44	70.60 ± 10.74	146.73 ± 19.87	66.77 ± 8.91	11.51 ± 1.30	0.00
	Uterus	8.60 ± 0.74	9.54 ± 0.88	35.62 ± 2.90	8.08 ± 1.13	0.00	0.00	0.00
	Ovary	5.97 ± 0.80	8.92 ± 0.63	24.97 ± 2.10	8.80 ± 1.10	6.30 ± 0.98	0.00	0.00
	Intestine	21.73 ± 2.65	83.46 ± 11.14	130.28 ± 19.34	43.59 ± 3.71	13.44 ± 1.34	8.58 ± 1.31	0.00

Naringenin	Heart	3.23 ± 0.34	8.60 ± 0.87	10.44 ± 0.94	18.90 ± 1.48	37.96 ± 3.42	95.36 ± 10.72	14.82 ± 1.66
	Liver	14.98 ± 1.42	26.78 ± 2.39	40.47 ± 1.03	121.58 ± 11.64	616.85 ± 60.73	413.12 ± 50.73	15.99 ± 1.67
	Spleen	8.09 ± 1.07	12.22 ± 2.12	15.01 ± 2.10	83.01 ± 10.49	69.71 ± 6.26	111.57 ± 13.09	15.14 ± 8.78
	Lung	7.67 ± 2.00	26.33 ± 7.58	37.52 ± 9.28	54.69 ± 5.73	61.63 ± 4.50	94.13 ± 9.13	10.88 ± 5.77
	Kidney	2.91 ± 0.35	8.62 ± 0.78	54.76 ± 4.29	41.12 ± 3.67	47.99 ± 5.03	175.34 ± 12.62	5.08 ± 0.59
	Brain	3.96 ± 0.40	9.95 ± 1.05	5.91 ± 0.78	13.22 ± 1.36	11.52 ± 1.53	18.79 ± 2.11	0.00
	Stomach	136.19 ± 16.40	164.32 ± 14.87	255.32 ± 19.99	393.85 ± 35.16	323.57 ± 33.94	210.95 ± 15.19	0.00
	Uterus	10.67 ± 1.07	17.14 ± 1.80	99.58 ± 13.09	47.72 ± 4.90	120.04 ± 15.89	100.46 ± 11.27	0.00
	Ovary	6.55 ± 0.45	9.46 ± 0.55	19.22 ± 1.78	58.95 ± 4.08	67.62 ± 5.38	87.30 ± 8.30	0.00
	Intestine	57.06 ± 4.18	84.62 ± 4.79	142.69 ± 17.30	391.10 ± 44.99	565.62 ± 45.04	499.71 ± 47.52	0.00

Kaempferol	Liver	0.00	0.00	7.75 ± 1.12	37.65 ± 2.13	43.61 ± 5.29	10.18 ± 1.17	0.00
	Spleen	0.00	13.30 ± 1.51	199.66 ± 23.11	25.68 ± 2.71	24.49 ± 2.99	16.70 ± 1.08	0.00
	Lung	12.21 ± 1.28	22.19 ± 2.73	32.75 ± 4.10	15.20 ± 1.15	5.19 ± 0.81	3.52 ± 1.14	0.00
	Kidney	0.66 ± 0.06	20.79 ± 2.41	38.34 ± 4.51	67.31 ± 5.19	29.74 ± 3.31	0.00	0.00
	Stomach	7.46 ± 0.75	20.55 ± 2.16	24.88 ± 3.27	34.72 ± 3.56	17.44 ± 2.31	2.73 ± 0.31	0.00
	Uterus	3.03 ± 0.27	6.23 ± 0.72	21.54 ± 2.53	15.86 ± 1.22	11.30 ± 1.26	5.64 ± 0.63	0.00
	Intestine	25.24 ± 1.48	54.96 ± 5.09	159.55 ± 11.04	120.50 ± 9.60	98.37 ± 9.36	46.17 ± 3.34	0.00

## Data Availability

No data were used to support this study.

## Abbreviations

UPLC-MS/MS: Ultra performance liquid chromatography tandem mass spectrometry
TFDR: Total flavonoids from *Drynariae Rhizoma*
MRM: Multiple reactions monitoring
IS: Internal standard
ESI+: Positive ion mode of electrospray ionization
ACN: Acetonitrile
MeOH: Methanol
QC: Quality control
LOD: Limit of detection
LOQ: Limit of quantitation
S/N: Signal-to-noise ratio
RSD: Relative standard deviations
RE: Relative error.
